# Prevalence and patterns of pre-existing multimorbidity in pregnancy in Northern Ireland: a population-based, retrospective study using linked routinely collected healthcare data

**DOI:** 10.1186/s12884-025-07771-1

**Published:** 2025-06-07

**Authors:** Lisa Kent, Siang Ing Lee, Megha Singh, Steven Wambua, Katherine Phillips, Utkarsh Agrawal, Amaya Azcoaga-Lorenzo, Colin McCowan, Jonathon Kennedy, Holly Hope, Ngawai Moss, Rachel Plachcinski, Catherine Nelson-Piercy, Mairead Black, Sinead Brophy, Aideen Maguire, Dermot O’Reilly, Krishnarajah Nirantharakumar, Kelly-Ann Eastwood, The MuM-PreDiCT Group

**Affiliations:** 1 Centre for Public Health, Queen’s University Belfast, Belfast, UK; 2 Centre for Public Health, Administrative Data Research Centre Northern Ireland, Queen’s University Belfast, Belfast, UK; 3 Department of Applied Health Sciences, College of Medicine and Health, University of Birmingham, Edgbaston, Birmingham, UK; 4 Division of Population and Behavioural Sciences, School of Medicine, University of St Andrews, St Andrews, UK; 5 Centro de Salud Los Pintores, Instituto de Investigación Sanitaria Fundación Jiménez Diaz, Madrid, Spain; 6 Centre for Population Health and Wellbeing, Swansea University, Swansea, UK; 7 Division of Psychology and Mental Health, School of Health Sciences, Faculty of Biology Medicine & Health, Centre for Women’s Mental Health, The University of Manchester, Manchester, UK; 8 Patient and Public Representative, Birmingham, UK; 9 Guy’s and St, Thomas’ NHS Foundation Trust, London, UK; 10 School of Medicine, Medical Science and Nutrition, Aberdeen Centre for Women’s Health Research, University of Aberdeen, Aberdeen, UK; 11 Fetal Medicine Unit, St Michael’s Hospital, University Hospitals Bristol and Weston NHS Foundation Trust, Bristol, UK

**Keywords:** Maternal, Pregnancy, Multimorbidity, Long-term condition, Mental health, Obesity, Preconception health, Population health, Routinely collected data, Administrative data, Northern Ireland, United Kingdom

## Abstract

**Background:**

Multimorbidity in pregnancy increases health risks to women and babies, and creates challenges for services. The aim of this study was to explore the prevalence and patterns of maternal multimorbidity in a UK population.

**Methods:**

This population-based, retrospective study used individual-level, linked, routinely collected health data accessed via The Health and Social Care Northern Ireland Business Service Organisation Honest Broker Service within a Trusted Research Environment following the Five Safes Framework. Pregnancy episodes were ascertained from the Northern Ireland Regional Maternity Service Database and linked via unique Health and Care Number to secondary care diagnoses and primary care medications. Yearly prevalence (2012–2020) of multimorbidity (≥ 2 physical or mental health conditions) and complex multimorbidity (involvement of ≥ 3 organ systems) were calculated for the full cohort and stratified by age, deprivation, body mass index (BMI) and gravida. Cross-sectional analyses of prevalence and exploration of unique combinations of conditions and organ system involvement across strata were performed during a period of stability in detection rates (2014–2019).

**Results:**

The annual number of pregnancies ranged from *n* = 24,403 (2012) to *n* = 19,504 (2020). Prevalence of maternal multimorbidity ranged from 18.2% (95%CI: 17.7–18.7%) (2012) to 22.8% (95% CI: 22.3–23.4%) (2016) and mostly involved coexistence of physical and mental health conditions (range: 13.0–17.4%). Complex multimorbidity ranged from 4.0% (2012) to 6.1% (2017). The mental health system demonstrated the highest prevalence compared to all other organ systems (range: 18.6–26.2%).

Multimorbidity was higher at extremes of maternal age (< 25y:24.15%; 25-34y:21.20%; ≥ 35y:23.39%), and increased with deprivation (least deprived:19.61%; most deprived:25.78%), BMI (healthy:18.37%; obesity III:39.18%), and gravida (first pregnancy:19.18%; ≥ 5 pregnancies:30.69%). Mental health multimorbidity most impacted the youngest group (< 25y:4.60%; 25-34y:1.36%; ≥ 35y:0.85%) and those who were underweight (3.73% vs 1–2% in other categories).

Mental health represented the most common organ system involved in multimorbidity (18.6% of the total study population), followed by respiratory (7.3%) and dermatology (7.2%).

**Conclusions:**

Multimorbidity impacts over 1 in 5 pregnant women in NI, with complex multimorbidity affecting over 1 in 20. This may present challenges across public health, primary and community care and maternity services which offer support to women with multimorbidity throughout their reproductive journeys, from preconception through to long-term postnatal follow-up.

**Supplementary Information:**

The online version contains supplementary material available at 10.1186/s12884-025-07771-1.

## Background

The co-existence of two or more long-term conditions affecting either physical or mental health, referred to as multimorbidity, has a significant impact on individuals, their families and health systems, and increases the likelihood of polypharmacy, disability and mortality [1, 2]. It is increasingly recognised that multimorbidity in pregnancy presents increased health risks to women and their babies. Previous work has shown an increased risk of preterm birth in women with two or more conditions, increasing further for those with four or more conditions [3]. There are also associated challenges for care of women throughout their reproductive journey, including the coordination of services for managing individual conditions, leading to increased healthcare utilisation and costs [4, 5].

Estimates of prevalence in maternal multimorbidity have varied across the four UK nations, reflecting the separate routinely collected healthcare databases used by the MuM-PreDiCT UK Consortium: CPRD GOLD, covering four percent of GP practices in England, Scotland, Wales and Northern Ireland (NI); the SAIL databank covering the whole population of Wales; and Scottish Morbidity Records from two Scottish regional health boards [6]. Primary care data and prescribed medication data from the CPRD GOLD and SAIL cohorts estimates maternal multimorbidity to be around 44 and 46%, respectively [6]. In contrast, a Scottish cohort using secondary care data and community medications estimated prevalence of multimorbidity to be 20% [6].

Given that NI differs from other UK nations in some key characteristics, it is important to explore and better understand the epidemiology of maternal multimorbidity in the region. Socioeconomic deprivation is higher in NI than other UK nations [7], while the health and care system is also reported by the British Medical Association to underperform compared to other UK regions [8, 9]. Previous research has also highlighted that the mental health burden in NI is higher than other UK countries [10]. These factors alone have the potential to translate into differences in population health, however there is further evidence that maternal health in NI differs from other regions. For example, maternal obesity, which is associated with increased rates of multimorbidity, has been shown to affect 22.5% of pregnant women in NI [11]. This represents a higher proportion compared to UK-wide estimates (19.1% in 2018), and pooled global (16.3%) and European (14.6%) estimates in the decade 2010–2019 [6, 12]. Additionally, whole population data is available for NI, which negates the need to rely on only a four percent sample from CPRD GOLD.

The primary aim of this study was to explore the prevalence and patterns of maternal multimorbidity using population-wide data for all women who gave birth in NI. We also had a number of secondary aims: (1) to explore underlying temporal trends in demographic, socioeconomic and health-related risk factors which may influence prevalence of multimorbidity; (2) to estimate the prevalence and patterns of multimorbidity and complex multimorbidity within pregnant women stratified by maternal age, deprivation, body mass index (BMI) and gravidity, and (3) to explore the utility of NI’s healthcare datasets for detecting health conditions and multimorbidity over time (2012–2020, inclusive) (Appendix 1; Supplementary Material).

## Methods

This population-based, retrospective study of prevalence and patterns of multimorbidity in pregnancy was designed to align with the methods of previous MuM-PreDiCT studies to allow for comparison between UK nations.

### Data source and Linkage

The Health and Social Care Northern Ireland Business Service Organisation Honest Broker Service (HBS) performed linkage between a number of datasets and provided researcher access to de-identified datasets within a Trusted Research Environment (TRE). Pregnancy episodes were ascertained from the Northern Ireland Regional Maternity Service Database (NIMATS). Individual-level data from other routinely collected healthcare databases were linked on a one-to-one basis via unique Health and Care Number (HCN) to individuals with records in NIMATS. The Patient Administration System (PAS) was used to ascertain admissions to secondary care hospitals and associated dates and ICD-10 codes for primary and secondary diagnoses. The Family Practitioner Services Enhanced Prescribing Database (EPD) was used to ascertain medications prescribed in Primary Care and dispensed in the community, and for each item the date of issue and eight-digit British National Formulary (BNF) code. Maternal postcode was linked to the NI Multiple Deprivation Measure (NIMDM) to give an indication of area-level deprivation for the mother’s residence at the time of pregnancy [13]. The NIMDM is a measure of relative deprivation within the NI population, and is a composite of seven domains including income, employment, health, education, access to services, living environment and crime. All personal identifiers were removed from the data to ensure non-identifiability prior to researcher access. In addition, all outputs were screened by HBS staff before release to ensure confidentiality following the Five Safes Framework [14].

### Cohort selection

The population spine for the NI cohort was defined as all pregnancies from all individuals with a valid HCN recorded in NIMATS. The start of each pregnancy was calculated from the variable “expected date of confinement”, estimated by ultrasound examination, minus 280 days. Pregnancies with a start date between 1^st^ January 2012 to 31^st^ December 2020 were retained. Pregnancies were included if the mother was aged between 15 to 49 years inclusive at the time of birth, aligning with previous analyses in other MuM-PreDiCT cohorts and reflecting the majority of reproductive age individuals.

### Identification of long-term conditions and multimorbidity

**Fig. 1:**
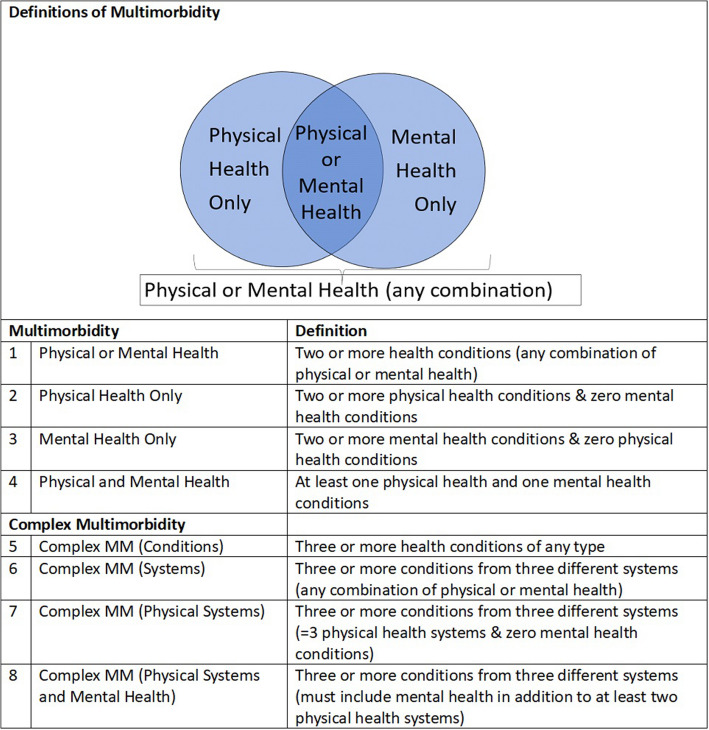
Definitions of multimorbidity and complex multimorbidity

Multimorbidity was defined as the presence of two or more physical or mental health conditions, and further stratified by physical health multimorbidity, mental health multimorbidity, and multimorbidity including both physical and mental health conditions (see Fig. 1)[6]. Complex multimorbidity was defined primarily as at least three conditions impacting at least three separate organ systems, in line with previous research in multimorbidity [15]. In order to compare multimorbidity rates in pregnancy with previous MuM-PreDiCT studies, a further definition of three or more conditions regardless of systems impacted was also used within the longitudinal trends analysis.

In this study, maternal pre-existing multimorbidity was ascertained using the MuM-PreDiCT phenome of 79 long-term physical and mental health conditions, including relapsing and remitting conditions [6]. Briefly, the phenome and associated definitions were developed via review of existing literature [16] and a Delphi process conducted with patient and public representatives, clinicians, researchers and data scientists [6]. For this study, the resultant 79 conditions were mapped to both secondary care admission ICD-10 diagnostic codes and, where applicable, patterns of community dispensed medications which likely indicated the presence of a condition of interest. Diagnostic codes were obtained from the PAS database and medications from the EPD database. Only conditions that were identified as pre-existing prior to the start date of each pregnancy were retained. The earlier work by the MuM-PreDiCT consortium for identification of long-term conditions via medications required at least four prescriptions in any 12-month period prior to the start of pregnancy. However, in NI, scan rates of prescription data to the EPD can vary, as previously described [17]. Therefore, in this NI study, the definition of conditions identified by prescription data was adjusted to require at least two prescriptions in any 12-month period.

### Covariates

Covariates were obtained from NIMATS and grouped as follows: (1) maternal age (categorised as < 25 years, 25 to 34, and ≥ 35 years), (2) area deprivation (derived from the mother’s post-code recorded at the first antenatal appointment, linked to NIMDM via super output area and aggregated to quintiles; Q1 most deprived through to Q5 least deprived), (3) BMI measured at the first antenatal appointment (categorised as underweight < 18.5 kg/m^2^, healthy weight 18.5 to 24.9 kg/m^2^, overweight 25 to 29.9 kg/m^2^, obese I 30 to 34.5 kg/m^2^, obese II 35 to 39.9 kg/m^2^, and obese III ≥ 40 kg/m^2^) and (4) gravidity (categorised as 1, 2, 3, 4, or ≥ 5 pregnancies including current pregnancy), (5) settlement type (urban: Belfast & Derry, rural: open countryside/small villages, intermediate: large villages through to large towns, outside NI/not recorded), (5) ethnicity (white, non-white, unknown), and (6) smoking status recorded at the first antenatal visit (non-smoker, ex-smoker, smoker, missing). Statistical disclosure control was assessed separately for each analysis, and further aggregation of the grouping of these characteristics was performed until the counts within all strata were at least n = 10 to protect confidentiality of individuals. Missing values were retained and not imputed.

### Data analysis

#### Maternal characteristics

Yearly trends in selected background characteristics for pregnant women were explored over the full study period, including, maternal age, deprivation quintiles, BMI category and gravidity. Initially, the frequency and prevalence for each category were calculated per year and visualised using line graphs. To explore intersecting characteristics over the study period, the following combinations were also explored using stacked bar plots: (i) age, BMI and gravida within each deprivation quintile; (ii) BMI within each intersecting age/deprivation group; (iii) gravida within each intersecting age/deprivation group.

#### Longitudinal trends in prevalence of maternal multimorbidity in NI

Estimated prevalence and 95% confidence intervals were calculated on a yearly basis from 2012 to 2020 and presented as the percentage of women entering pregnancy with pre-existing multimorbidity using the total number of pregnancies with a start date falling within each year as the denominator. Yearly prevalence was visualised using dot plots with 95% confidence intervals.

Prevalence of multimorbidity was first estimated using a full look-back period to the inception of each database. This was repeated with a standardised look-back period for each pregnancy episode. For detection of conditions through ICD-10 codes in the PAS database which has an inception date of 2006, the standardised look-back period was set at 5 years prior to the start of pregnancy. Whereas the standardised look-back period for EPD was set to 2 years due to the later inception date (2010).

To provide context to observed trends in yearly prevalence estimates of multimorbidity, we also explored the yearly prevalence of pre-existing health conditions categorised by organ system.

#### Maternal characteristics and multimorbidity in NI

Yearly prevalence of all subtypes of multimorbidity and complex multimorbidity was estimated within each age, deprivation, BMI and gravida group. To explore intersectionality of multimorbidity across characteristics, yearly prevalence of multimorbidity was also estimated for intersecting age and deprivation groups. For these stratified analyses, conditions were detected using a standardised look-back period of 5 years for PAS and 2 years for EPD.

#### Cross-sectional analysis of patterns of maternal multimorbidity

Cross-sectional analyses of patterns in maternal multimorbidity were limited to individuals with a pregnancy start date falling between 2014 and 2019, inclusive. This time period represents a period of stability in prevalence rates of multimorbidity in pregnancy as observed in the yearly prevalence analysis.

Maternal characteristics were categorised as described above and summarised as frequency and percentage for the full cohort, and for subgroups with (i) at least one long-term condition, (ii) multimorbidity (iii) physical health multimorbidity, (iv) mental health multimorbidity, (v) mental and physical health multimorbidity, and (vi) complex multimorbidity (3 or more systems).

Unique combinations of long-term conditions contributing to multimorbidity were detected and summarised as frequency and percentage of pregnancies impacted. The top 10 most prevalent combinations are reported for the full study cohort and stratified by age and deprivation. This was repeated to explore unique combinations of conditions impacting pregnant women within intersecting age and deprivation groups. Unique combinations of organ systems implicated in complex multimorbidity was also explored for the full study cohort, however small counts prevented further stratification by age and deprivation.

Prevalence of each organ system being implicated either on its own, in addition to at least one other organ system (multimorbidity), and in addition to at least two other organ systems (complex multimorbidity) was established and visualised using a histogram.

#### Sub-analysis: utility of different data sources for detecting conditions

See Appendix 1 in Supplementary Material.

All data preparation, analyses and visualisation were performed in R [18] [R citation].

## Results

### Summary maternal characteristics

The number of pregnancies per year in NI declined over the study period from 24,403 in 2012 to 19,504 in 2020. This reflects an underlying decline in general fertility rate from 67.5 live births per 1,000 (2012), to 58.5/1,000 (2020) [19]. Over the course of the study period, there were changes in the background characteristics of pregnant women; notably higher proportions of women were older (≥ 35 years: 20.6% to 24.1%), living with obesity (BMI ≥ 30: 18.9% to 26.1%), and had higher numbers of pregnancies (five or more pregnancies: 8.6% to 10.8%) (Fig S1 in Additional Figures).

When stratified by deprivation quintile, women from the most deprived areas demonstrated the largest increases in advancing age and obesity prevalence over time (Fig S2 in Additional Figures). When stratified by both deprivation and age (Fig S3 in Additional Figures), obesity prevalence in both the 25 to 34 years and ≥ 35 years old groups demonstrated disparity between the most and least deprived areas, which appeared to increase further over time. By 2020, approximately one in three pregnant women aged over 25 years and residing in deprived areas had co-morbid obesity (25-34y: 31.8%; ≥ 35y: 32.9%) compared to around one in five in the least deprived areas (25-34y: 19.5%; ≥ 35y: 21.2%). The proportion of women with three or more pregnancies did not demonstrate a distinct change over time in intersectional groups, however a higher proportion overall was observed in older groups in more deprived areas (Fig S4 in Additional Figures).

### Longitudinal trends in prevalence of maternal multimorbidity in NI

**Fig. 2:**
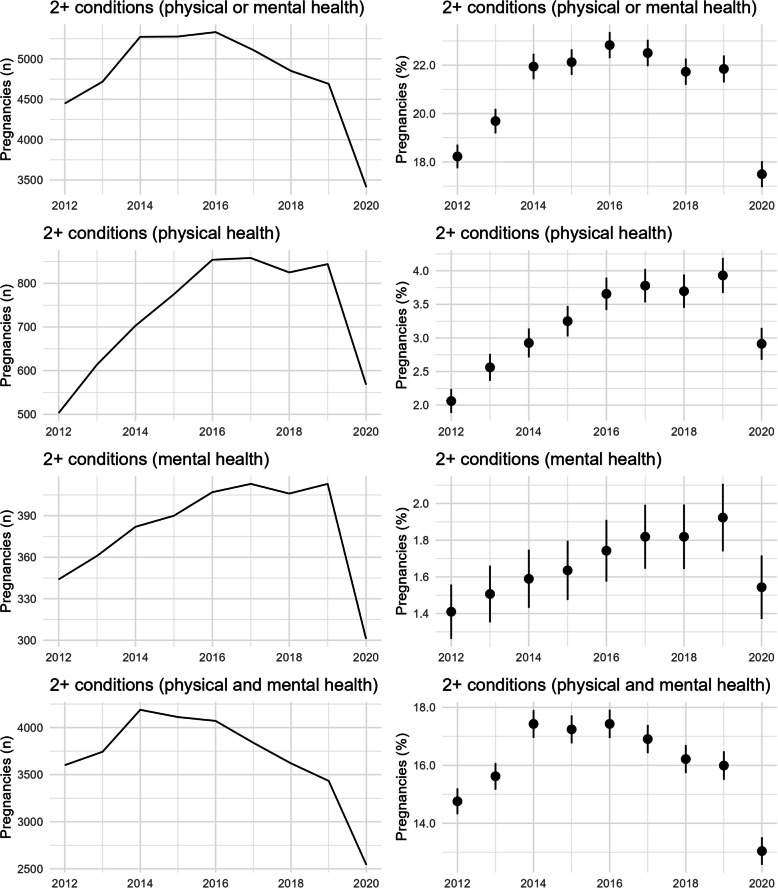
Temporal changes (2012 to 2020) in detectable multimorbidity using a full look-back period

**Fig. 3:**
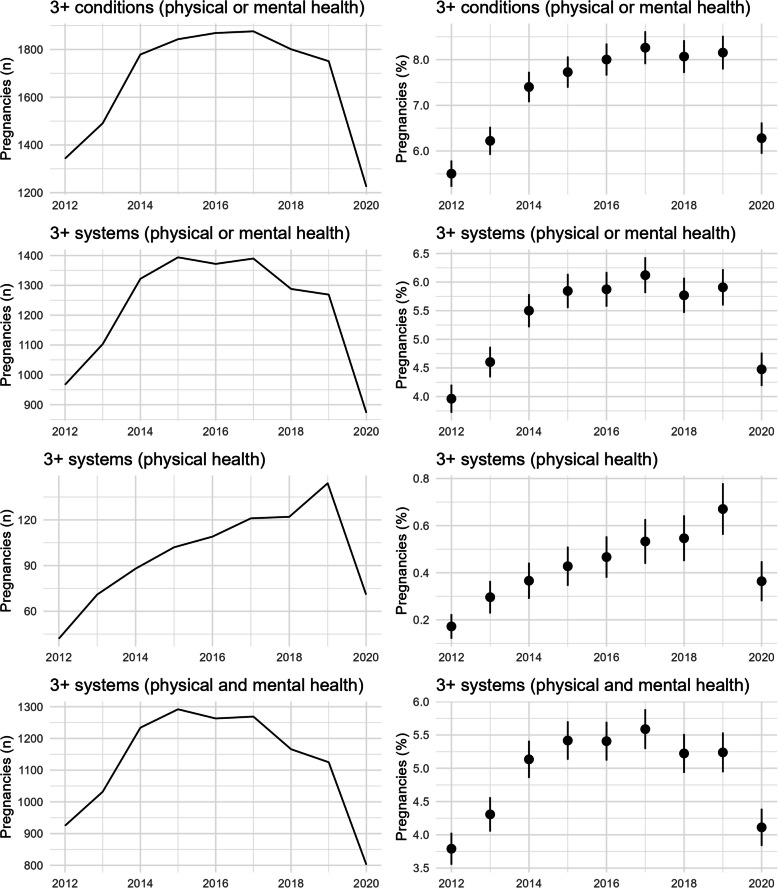
Temporal changes (2012 to 2020) in detectable complex multimorbidity using a full look-back period

Prevalence of pre-existing multimorbidity ranged from an early low of 18.2% (95%CI: 17.7 to 18.7%) in 2012, and peaking at 22.8% (95% CI: 22.3 to 23.4%) in 2016. There was an upward trend between 2012 and 2014 where confidence intervals are not overlapping, after which the prevalence plateaued at around 22% with overlapping confidence intervals in estimates for the years 2014 to 2019, before a significant decrease to the lowest rate of 17.5% (95%CI:17.0 to 18.0%) in 2020 (Fig. 2). These findings provided the rationale for limiting the subsequent cross-sectional analyses to the period of stability between 2014 to 2019. When multimorbidity rates were stratified by type, the majority of pregnant women had both physical and mental health conditions (range 13.0 to 17.4% over the study period), whereas fewer pregnancies had multimorbidity comprising of only physical health conditions (range 1.9 to 3.9%) or of only mental health conditions (range 1.4 to 1.9%). Complex multimorbidity (3 or more systems) ranged from 4.0% of women in 2012 to 6.1% in 2017 (Fig. 3).

Adopting a standardised look-back period resulted in lower prevalence across all time periods compared to a full look-back period to the inception of each database (Fig S5 in Additional Figures). However, a similar pattern of change was shown – multimorbidity increased from 11.0% to 12.4% between 2012 and 2014, then plateaued between 2014 to 2019, and declined in 2020 to 10.6%. Again, multimorbidity was comprised mainly of coexisting physical and mental health conditions. Prevalence of complex multimorbidity also appeared to increase between 2012 and 2014, plateauing thereafter, and falling in 2020 (Fig S6 in Additional Figures).

When individual conditions were grouped by organ system involvement and a standardised look-back period for detection used, the longitudinal trends in detectable rates varied (Fig S7 & S8 in Additional Figures). Mental health conditions demonstrated the highest annual rate across the study period compared to all other organ systems increasing from 18.6% of pregnancies in 2012 to 26.2% in 2019. Conditions affecting endocrine and respiratory systems also demonstrated an upwards trend, with endocrine conditions increasing from 3.0% to a peak of 4.4% in 2019, and respiratory conditions increasing from 7.4% in 2012 to a peak of 8.3% in 2019. Both systems demonstrate a decline in 2020, during which time the COVID-19 pandemic was emerging, with rates of pregnant women living with at least one pre-existing respiratory condition falling to 7.2%, and rates of pregnant women with at least one pre-existing endocrine condition falling to 4.2%. The percentage of pregnancies with at least one dermatological condition showed a markedly different trend, decreasing over the study period from 7.2% to 4.1%. Distinct patterns of change in detectable conditions were not observed in other organ systems.

### Annual prevalence of multimorbidity across maternal characteristics

The results for annual detectable rates of multimorbidity subtypes across maternal characteristic groups using standardised look-back periods are summarised in Table 1 and illustrated in full in Figures S9-S16 in Additional Figures.

**Table 1:** Summary of longitudinal trends in prevalence of maternal multimorbidity in NI between 2012 and 2020

	Group with highest prevalence of multimorbidity (MM) * clearly highest ~ highest but with overlap of CI				**Temporal trend** ↗ increasing MM over time ↘ decreasing MM over time = no clear trend				**Trajectory change in 2020** ↑ increased in 2020 ↓ decreased in 2020 = no clear change NA not able to explore			
**Type of Multimorbidity**	**PH or MH**	**PH only**	**MH only**	**PH + MH**	**PH or MH**	**PH only**	**MH only**	**PH + MH**	**PH or MH**	**PH only**	**MH only**	**PH + MH**
**Age (See Fig S9)**												
< 25y	*		*		** = **	↘	↗	** = **	↓	=	** = **	↓
25-34y					** = **	↘	=	** = **	↓	↓	** = **	↓
35 + y	*	*		*	** = **	** = **	=	** = **	↓	↓	↓	↓
**Deprivation (See Fig S11)**												
Q1 (most deprived	*		*	*	** = **	↘	=	** = **	=	=	=	=
Q2					** = **	↘	=	** = **	=	=	=	=
Q3		*			** = **	** = **	=	↗	↓	↓	=	↓
Q4		*			↗	** = **	=	↗	↓	↓	=	↓
Q5		*			** = **	** = **	=	** = **	↓	↓	=	=
**BMI Groups (See Fig S13)**												
< 18.5			*		** = **	** = **	=	** = **	NA	NA	NA	NA
18.5–24.9					** = **	** = **	=	** = **	NA	NA	NA	NA
25–29.9					** = **	** = **	=	** = **	NA	NA	NA	NA
30–34.9					** = **	** = **	=	** = **	NA	NA	NA	NA
35–39.9					** = **	** = **	=	** = **	NA	NA	NA	NA
≥ 40	*	*		*	** = **	** = **	=	** = **	NA	NA	NA	NA
**Gravida (See Fig S15)**												
1		** ~ **			** = **	↘	=	** = **	↓	↓	↓	↓
2		** ~ **			** = **	↘	=	** = **	↓	=	↓	↓
3		** ~ **			** = **	** = **	=	** = **	↓	=	=	↓
4		** ~ **			** = **	** = **	=	** = **	↓	=	=	↓
≥ 5	*	** ~ **	*	*	** = **	** = **	=	** = **	=	=	=	=

*MM* Multimorbidity, *MH* Mental Health, *PH* Physical Health

#### Age

Multimorbidity consisting of only physical conditions increased with age, with prevalence rates peaking at 4.6% in the oldest group (≥ 35y). Rates of physical-only multimorbidity were lowest in the youngest group, and also appeared to decrease over time, from 2.8% in 2012 to 1.1% in 2020 (Fig S9 in Additional Figures). Conversely, multimorbidity consisting of only mental health conditions most impacted the youngest age group, increasing over time with a peak of 3.3%, compared to other age groups which had rates consistently less than 1%. Differences across age groups in the combined physical and mental health multimorbidity group were less distinct, however the oldest group (≥ 35y) demonstrated a slightly higher prevalence of maternal multimorbidity (7.6 to 9.7%). Complex multimorbidity (3 or more systems) demonstrated a dose–response pattern, increasing across age groups (< 25y: 1.5 to 2.4%; 25-34y: 2.2 to 2.9%; ≥ 35y: 2.7 to 3.8%) (Fig S10 in Additional Figures).

#### Deprivation

Detection of multimorbidity was greater as deprivation increased, and this pattern was consistent across all years (Fig S11 in Additional Figures). However, when prevalence was stratified by type of condition, results indicate that higher rates of multimorbidity in the most deprived are driven by mental health conditions, which also increased from 1.2% in 2012 to 2.2% 2017, while physical-only multimorbidity decreased in this group from 3.2% to 2.0%. In contrast, multimorbidity consisting of only physical health conditions was higher in the *least* deprived areas, and consistent over much of the study period (range: 3.1 to 4.1%). The decline in rates of multimorbidity, particularly physical multimorbidity, between 2019 and 2020, were greatest in the least deprived group compared to the most deprived. There was a general trend of higher prevalence of complex multimorbidity in the most deprived group compared to the least deprived group, however, change over time was not clear (Fig S12 in Additional Figures).

#### Intersection of age and deprivation

**Fig. 4:**
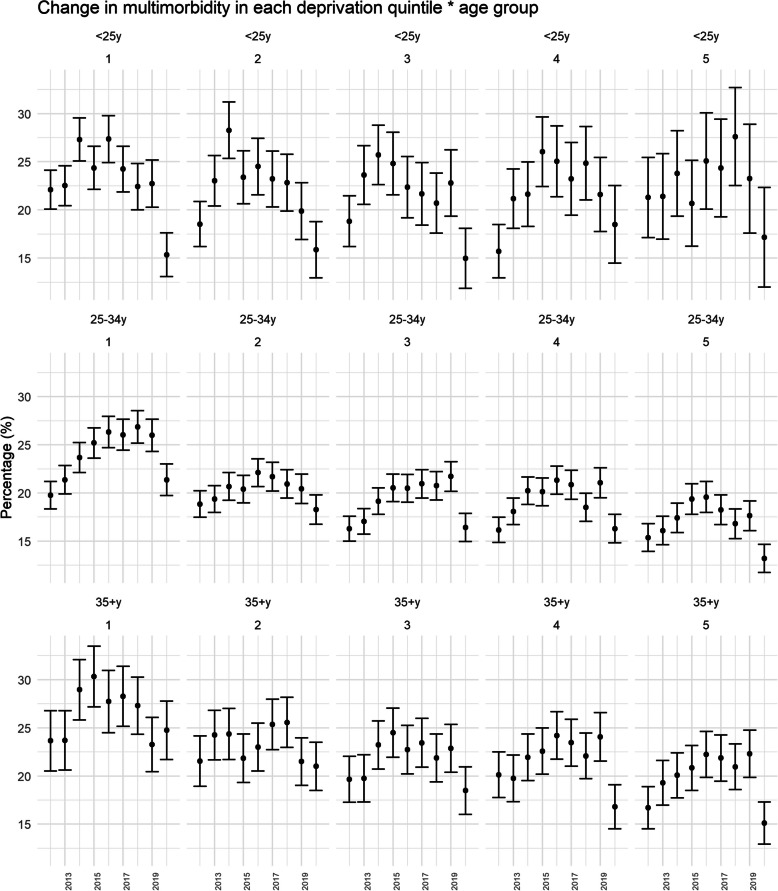
Temporal changes (2012 to 2020) in detectable multimorbidity across intersecting age and deprivation groups

Rates of multimorbidity in the youngest age group did not differ across deprivation quintiles and was broadly consistently between 20 to 25% in the time period 2012 to 2019 (Fig. 4). However, in the older age groups, differences were observed between deprivation quintiles, with prevalence estimates more than 25% in 25–34 year olds living in the most deprived areas, and up to 30% in those aged at least 35 years and living in the most deprived areas.

#### Maternal BMI

Multimorbidity increased as BMI class increased, but did not demonstrate any clear change over time (multimorbidity healthy BMI: 9.3% to 12.8% vs obesity III: 23.9 to 25.3%) (Fig S13 in Additional Figures). However, when stratified by type, mental health multimorbidity was demonstrably greater in women who were underweight (< 18.5 kg/m^2^). A dose–response was also observed for complex multimorbidity, with rates highest in the group with obesity class III (7.8 to 8.9%) (Fig S14 in Additional Figures).

#### Gravida

Multimorbidity detection increased with advancing number of pregnancies with rates of between 15.8 to 19.7% for multimorbidity, and 3.8 to 5.1% for complex multimorbidity in the group with five or more pregnancies (Fig S15 & S16 in Additional Figures). When stratified by type, women with five or more pregnancies also showed a significantly greater prevalence of mental health multimorbidity (1.4 to 1.9%) and of coexisting physical and mental health (10.6 to 14.8%). No clear differences between groups were observed for physical only multimorbidity, however, groups with one or two pregnancies showed declining detectable rates over time.

### Cross-sectional analysis of multimorbidity

#### Prevalence of preexisting multimorbidity in pregnancy

Cross-sectional demographic, socioeconomic and health-related characteristics are presented in Table 2 for the cohort of women with pregnancy start dates between January 2014 and December 2019 inclusive. In total, 22.2% of pregnant women in NI were entering pregnancy with multimorbidity, and 5.8% with complex multimorbidity. Multimorbidity comprised mainly of coexisting physical and mental health conditions (16.9%), with 3.5% living with physical-only multimorbidity, and 1.8% living with mental-health-only multimorbidity. Multimorbidity was higher at extremes of maternal age (< 25y: 24.1%; 25-34y: 21.2%; ≥ 35y:23.4%), and higher in the white ethnicity group (22.6% vs 8.64%). Multimorbidity also increased as deprivation increased, with a larger step increase moving into the most deprived areas (least deprived: 19.6%; most deprived: 25.8%). Interestingly, mental health-only multimorbidity increased with increasing deprivation, whilst physical-only multimorbidity increased as deprivation *decreased.* Multimorbidity was higher in urban (23.6%) and intermediate (23.5%) settlements in comparison to rural areas (18.8%). Again, divergent patterns were apparent in mental health morbidity, which was higher in the urban group (2.7% vs 1.9% and 0.81%), in contrast to physical morbidity which was higher in the rural group (4.3% vs 2.7% and 3.4%). A gradual increase in multimorbidity across BMI class was observed from 18.4% in the healthy BMI class to 39.2% in those with obesity class III. Multimorbidity also increased with increasing number of pregnancies (first pregnancy: 19.2%; ≥ 5 pregnancies: 30.7%) with mental health morbidities featuring more prevalently as the number of pregnancies increased. Prevalence of multimorbidity was higher in those who continued to smoke during pregnancy (33.6%) and those who had previously smoked (25.8%) compared to non-smokers (18.8%).

**Table 2:** Demographic, socioeconomic and health-related characteristics of women within the study cohort (2014–2019)

	**Full Cohort**		**Women with at least one long-term condition**		**Women with at least 2 long-term conditions**		**Women with at least 2 physical health conditions (& no mental health)**		**Women with at least 2 mental health conditions (& no physical)**		**Women with at least 1 physical and 1 mental health condition**		**Women with at least 3 conditions from 3 different systems**	
	**137,750**		**77,733 (56.43%)**		**30,535 (22.17%)**		**4,859 (3.53%)**		**2,411 (1.75%)**		**23,265 (16.89%)**		**8,035 (5.83%)**	
	**n**	**Col %**	**n**	**Row %**	**n**	**Row %**	**n**	**Row %**	**n**	**Row %**	**n**	**Row %**	**n**	**Row %**
**Age**														
< 25y	21,609	15.69	13,517	62.55	5219	24.15	393	1.82	993	4.60	3833	17.74	976	4.52
25-34y	84,346	61.23	46,146	54.71	17,880	21.20	2962	3.51	1148	1.36	13,770	16.33	4782	5.67
≥ 35y	31,795	23.08	18,070	56.83	7436	23.39	1504	4.73	270	0.85	5662	17.81	2277	7.16
**Ethnicity**														
Non-white/missing	4662	3.38	1421	30.48	403	8.64	130	2.79	24	0.51	249	5.34	97	2.08
White	133,088	96.62	76,312	57.34	30,132	22.64	4729	3.55	2387	1.79	23,016	17.29	7938	5.96
**Deprivation**														
1	29,439	21.37	18,535	62.96	7588	25.78	743	2.52	910	3.09	5935	20.16	1939	6.59
2	29,226	21.22	16,543	56.6	6453	22.08	920	3.15	577	1.97	4956	16.96	1772	6.06
3	28,151	20.44	15,443	54.86	6056	21.51	1101	3.91	392	1.39	4563	16.21	1567	5.57
4	27,175	19.73	14,881	54.76	5823	21.43	1132	4.17	303	1.11	4388	16.15	1625	5.98
5	22,617	16.42	11,846	52.38	4435	19.61	942	4.17	203	0.90	3290	14.55	1091	4.82
NA	1142	0.83	485	42.47	180	15.76	21	1.84	26	2.28	133	11.65	41	3.59
**Settlement Type**														
urban	26,901	19.53	15,924	59.19	6335	23.55	736	2.74	713	2.65	4886	18.16	1564	5.81
rural	38,224	27.75	19,584	51.23	7190	18.81	1652	4.32	310	0.81	5228	13.68	1855	4.85
intermediate	71,483	51.89	41,740	58.39	16,830	23.54	2450	3.43	1362	1.91	13,018	18.21	4575	6.40
not recorded/outside NI	1,142	0.83	485	42.47	180	15.76	21	1.84	26	2.28	133	11.65	41	3.59
**BMI Class**														
< 18.5 kg/m^2^	2495	1.81	1420	56.91	479	19.20	51	2.04	93	3.73	335	13.43	90	3.61
18.5–25 kg/m^2^	61,772	44.84	31,704	51.32	11,349	18.37	1934	3.13	1114	1.80	8301	13.44	2527	4.09
25–30 kg/m^2^	41,225	29.93	23,415	56.80	9075	22.01	1498	3.63	671	1.63	6906	16.75	2363	5.73
30–35 kg/m^2^	18,821	13.66	11,937	63.42	5216	27.71	794	4.22	309	1.64	4113	21.85	1529	8.12
35–40 kg/m^2^	8109	5.89	5555	68.50	2583	31.85	357	4.40	140	1.73	2086	25.72	830	10.24
> 40 kg/m^2^	3874	2.81	2887	74.52	1518	39.18	184	4.75	54	1.39	1280	33.04	602	15.54
NA	1454	1.06	815	56.05	315	21.66	41	2.82	30	2.06	244	16.78	94	6.46
**Gravida**														
1	43,185	31.35	22,399	51.87	8281	19.18	1502	3.48	698	1.62	6081	14.08	2051	4.75
2	40,708	29.55	22,254	54.67	8478	20.83	1390	3.41	628	1.54	6460	15.87	2175	5.34
3	26,604	19.31	15,464	58.13	6059	22.77	973	3.66	433	1.63	4653	17.49	1612	6.06
4	13,947	10.12	8665	62.13	3633	26.05	533	3.82	289	2.07	2811	20.15	974	6.98
5plus	13,306	9.66	8951	67.27	4084	30.69	461	3.46	363	2.73	3260	24.50	1223	9.19
**Smoking Status**														
Currently smoking	18,848	13.68	13,779	73.11	6335	33.61	327	1.73	1108	5.88	4900	26.00	1542	8.18
Ex-smoker	13,812	10.03	8647	62.6	3564	25.8	402	2.91	333	2.41	2829	20.48	1009	7.31
Non-smoker	48,696	35.35	24,371	50.05	9171	18.83	2167	4.45	389	0.8	6615	13.58	2470	5.07
Not recorded	56,394	40.94	30,936	54.86	11,465	20.33	1963	3.48	581	1.03	8921	15.82	3014	5.34

The highest rates of multimorbidity were observed in the group with obesity class III (39.2%), obesity class II (31.9%), current smokers (33.6%), and those with gravidity of 5 or more (30.7%). This cross-sectional analysis also demonstrated highest rates of mental health multimorbidity in those aged under 25 years (4.6%), in those classed as underweight (3.7%), and in the most deprived areas (3.1%). The groups living with obesity class III were most impacted by complex multimorbidity (15.5%). This was followed by those with obesity class II (10.2%), and women with five or more pregnancies (9.2%).

#### Unique combinations of multimorbidity in pregnant women

The top ten unique combinations of maternal multimorbidity and complex multimorbidity primarily featured CMHD and atopic conditions such as asthma, eczema and allergic rhinoconjunctivitis (Table S1 & S2 in Additional Tables). At population level, the most common combination was CMHD and asthma (2.3%), and this persisted as the most prevalent combination in many of the stratified groups (Tables S3 & S4 in Additional Tables). When stratified by age, the youngest group showed unique combinations which featured a number of mental health conditions including CMHD, serious mental illness and other mental health conditions (including obsessive compulsive disorder, personality disorder, dissociative disorder or self-harm/suicide ideation) (Table S3 in Additional Tables). This predominance of a wide range of mental health conditions persisted in the younger age groups when further stratified by deprivation, appearing in the top 10 combinations in young women in both the most deprived and most affluent areas (Table 3). When unique combinations were explored within the older age group (aged ≥ 35y), women living in the most deprived areas showed higher rates of common mental health disorders and conditions including gall stones and hypertension.

**Table 3:** Top 10 unique combinations of maternal multimorbidity, stratified by age and deprivation (2014–2019)

	Most deprived & Age < 25y *n* = 7,757			Most deprived & Age 25–34y *n* = 16,774			Most deprived & Age 35 + y *n* = 4,908		
Rank	Conditions	n	%	Conditions	n	%	Conditions	n	%
1	CMHD + asthma	250	3.22	CMHD + asthma	443	2.64	CMHD + eczema	118	2.40
2	CMHD + eczema	191	2.46	CMHD + eczema	383	2.28	CMHD + asthma	104	2.12
3	CMHD + other MH	146	1.88	CMHD + other MH	149	0.89	CMHD + thyroid disorder	53	1.08
4	CMHD + migraine	67	0.86	CMHD + migraine	125	0.75	CMHD + allergic rhinoconjunctivitis	35	0.71
5	CMHD + asthma + eczema	63	0.81	CMHD + IBD	117	0.70	CMHD + IBD	32	0.65
6	SMI + other MH	58	0.75	CMHD + thyroid disorder	104	0.62	CMHD + migraine	25	0.51
7	CMHD + IBD	46	0.59	CMHD + Gall Stones	92	0.55	CMHD + gall Stones	20	0.41
8	depression* + other MH	37	0.48	CMHD + allergic rhinoconjunctivitis	84	0.50	CMHD + hypertension	17	0.35
9	SMI + asthma	33	0.43	CMHD + asthma + eczema	83	0.49	CMHD + endometriosis	16	0.33
10	CMHD + NDD	29	0.37	SMI + asthma	65	0.39	CMHD + asthma + eczema	16	0.33
	Least deprived & Age < 25y *n* = 1,746			Least deprived & Age 25–34y *n* = 14,047			Least deprived & Age 35 + y *n* = 6,824		
Rank	Conditions	n	%	Conditions	n	%	Conditions	n	%
1	CMHD + asthma	58	3.32	CMHD + eczema	271	1.93	CMHD + eczema	132	1.93
2	CMHD + eczema	51	2.92	CMHD + asthma	250	1.78	CMHD + asthma	120	1.76
3	CMHD + other MH	28	1.6	CMHD + thyroid disorder	109	0.78	CMHD + thyroid disorder	96	1.41
4	CMHD + migraine	13	0.74	CMHD + IBD	89	0.63	CMHD + allergic rhinoconjunctivitis	46	0.67
5	CMHD + asthma + eczema	13	0.74	CMHD + allergic rhinoconjunctivitis	84	0.60	CMHD + migraine	38	0.56
6	CMHD + IBD	11	0.63	CMHD + migraine	82	0.58	CMHD + IBD	34	0.5
7	SMI + other MH	11	0.63	asthma + eczema	78	0.56	CMHD + infertility	24	0.35
8	**	**	**	CMHD + asthma + eczema	60	0.43	Asthma + eczema	24	0.35
9	**	**	**	asthma + allergic rhinoconjunctivitis	56	0.40	Asthma + allergic rhinoconjunctivitis	24	0.35
10	**	**	**	CMHD + other MH	46	0.33	CMHD + endometriosis	23	0.34

*CMHD* = *Common Mental Health Disorder; SMI* = *Serious Mental Illness (may include bipolar disorder, schizophrenia, affective psychosis or non-affective psychosis); NDD* = *Neurodevelopmental Disorders (may include attention deficit hyperactivity disorder, autism, or learning difficulties); Other MH* = *Other Mental Health Condition (may include obsessive compulsive disorder, personality disorder, dissociative disorder or self-harm/suicide ideation); IBD* = *Irritable Bowel Disease*

^**^ Suppressed due to small counts in the least deprived & aged under 25-year-old group. CMHD, SMI, NDD, asthma and gall stones feature in combinations ranked 8, 9, 10 (combined count of n = 25 and rate of 1.43%)

#### Organ systems involved in multimorbidity in pregnant women

**Fig. 5:**
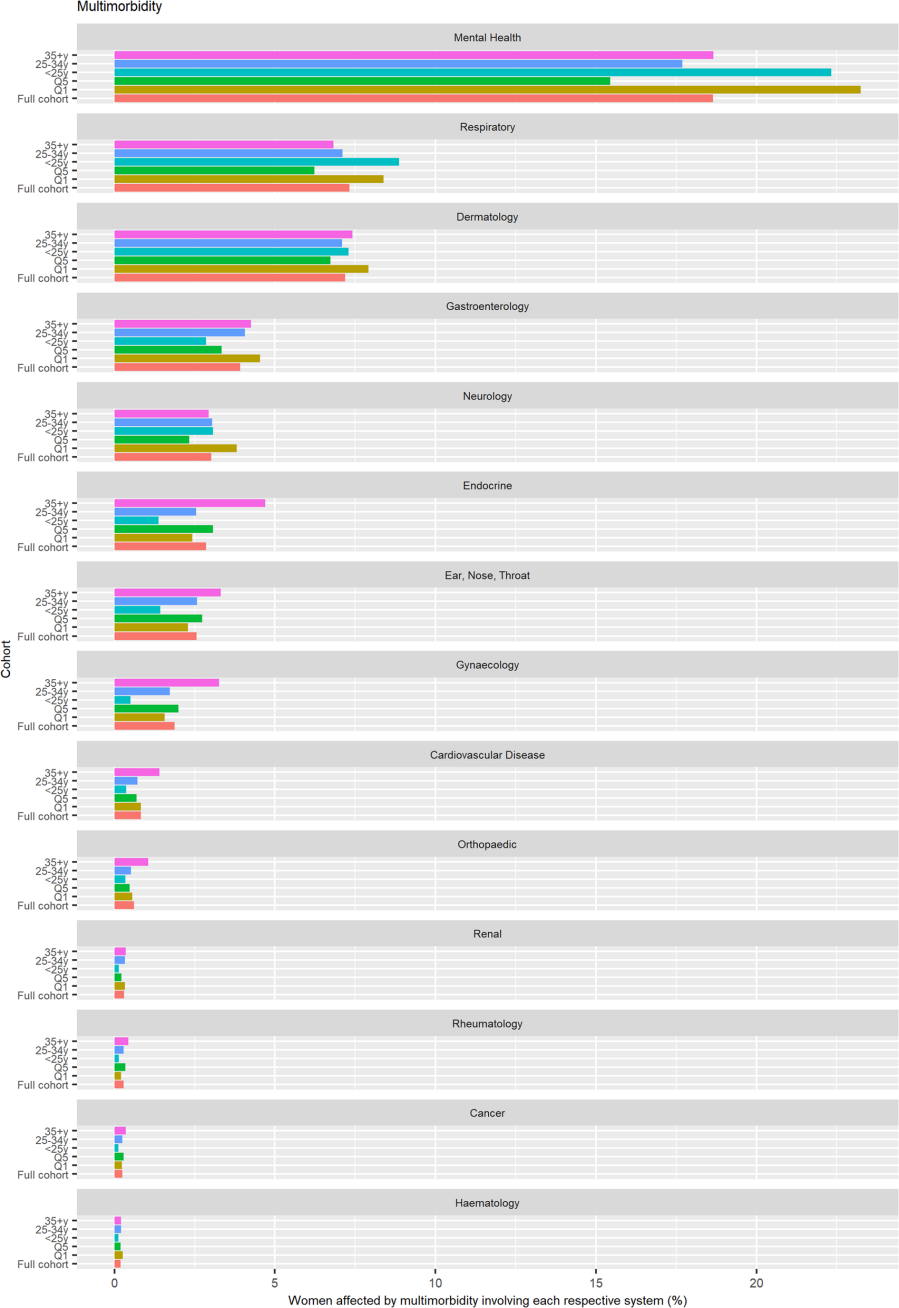
Organ systems involved in multimorbidity (detected using full look-back period; denominator: all pregnancies 2014–2019)

**Fig. 6:**
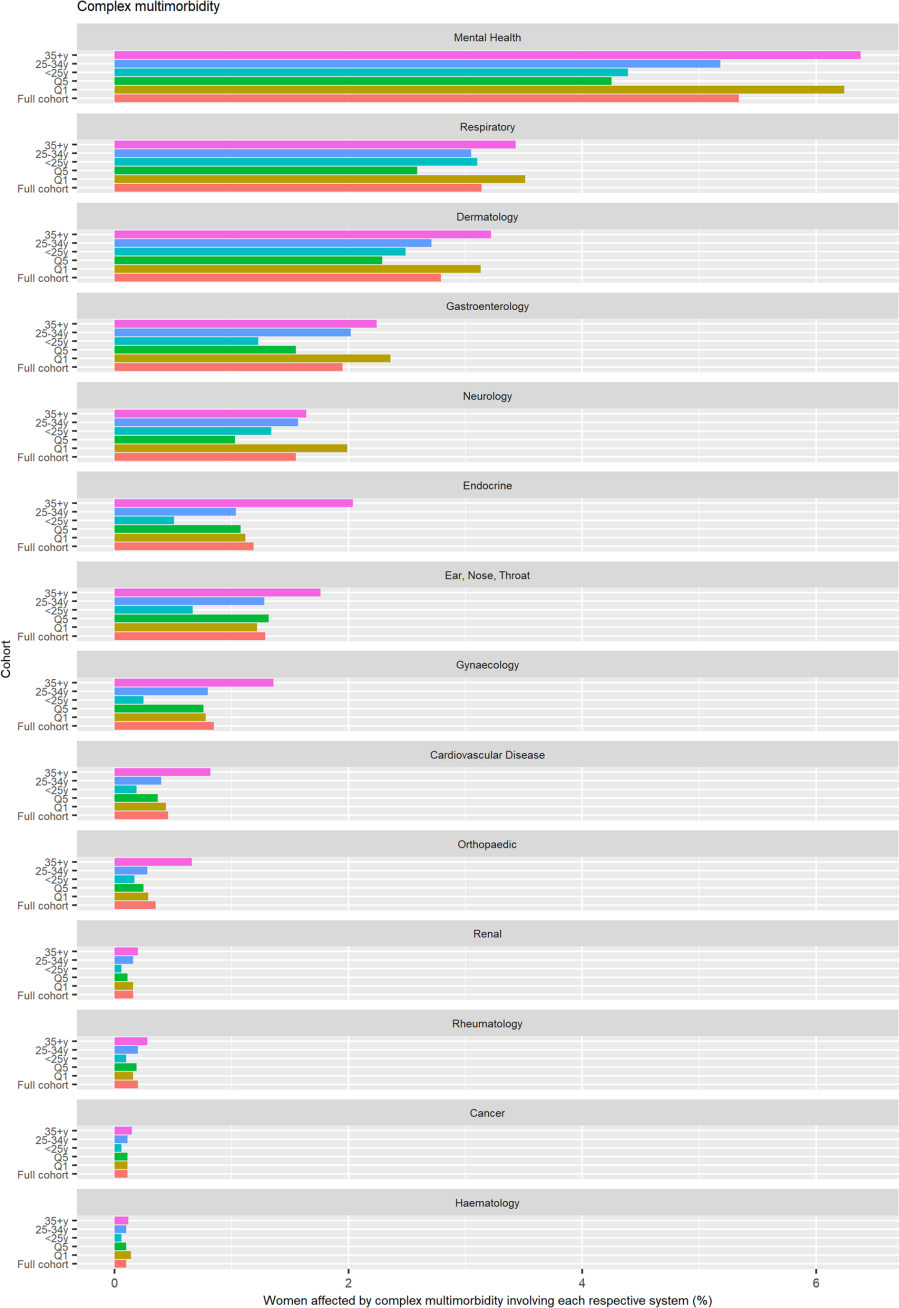
Organ systems involved in complex multimorbidity (detected using full look-back period; denominator: all pregnancies 2014–2019)

When each organ system was explored separately, mental health featured predominantly, with 41.9% of the total pregnant population presenting with a mental health condition either in isolation or in combination with other conditions (Fig S18 in Additional Figures). When exploring organ system involvement in multimorbidity, 18.6% of the total study population were living with a mental health condition in combination with at least one other condition (Fig. 5). The next most prevalent organ system implicated in multimorbidity was respiratory (7.3% of total study population), followed by dermatology (7.2%), gastroenterology (3.9%), neurology (3.0%), endocrine (2.9%), eyes, nose and throat (ENT) (2.6%), gynaecology (1.9), and cardiovascular (0.8%). A similar hierarchy of organ system involvement was observed for complex multimorbidity (Fig. 6).

When stratified by age, mental health conditions and respiratory conditions were implicated more often in multimorbidity in the youngest age group compared to the other age groups **(**Fig. 5**)**, whereas when looking at complex multimorbidity, mental health conditions were implicated more in the oldest age group (Fig. 6). The remaining organ systems featured more often in multimorbidity and complex multimorbidity in the oldest group (aged ≥ 35y). Most organ systems showed a greater prevalence in the most deprived areas, with the exception of endocrine, ENT, gynaecology.

### Sub-analysis: utility of different sources in detecting conditions

See Appendix 1 in Supplementary Material.

## Discussion

This is the first study to estimate the prevalence and patterns of multimorbidity in the entire population of pregnant women in NI, the most deprived nation in the UK. Over 22% of pregnant women in NI have at least two pre-existing health conditions. This mainly comprised of coexisting physical and mental health conditions (17%). Around 5% of women enter pregnancy with complex multimorbidity impacting at least three organ systems. With an estimated 20 thousand pregnancies per year, this means the NI maternity system is faced with over 1,000 births per year, 80 per month, 20 per week and approximately 2–3 per day in mothers who are living with complex multimorbidity.

In-depth exploration of multimorbidity sub-types and unique combinations of conditions provides information on the mosaic of patterns of multimorbidity across demographic and socioeconomic groups. This provides important insights, for instance, that multimorbidity is highest in the most deprived group and appears to be driven by mental health conditions. Younger women and underweight women have higher rates of multimorbidity comprised exclusively by mental health conditions (4.6% and 3.7% respectively). The youngest age group, particularly those in the most deprived areas, also demonstrate a relatively higher rate of serious mental illness, neurodevelopmental disorders and other mental health conditions.

Over the course of the study (2012–2020), our data demonstrate a change in the background demographic and socioeconomic characteristics of pregnant women living in NI. In particular, the annual number of pregnancies fell by 20% overall, with fewer younger women (< 25 years) entering pregnancy. Simultaneously, obesity rates increased over the study period (18.9% in 2012 to 26.1% in 2020), reflecting previous published evidence from NI [11]. This increase in maternal obesity prevalence is concerning, as the highest rates of multimorbidity (39.2%) and complex multimorbidity (15.5%) are seen in pregnant women living with obesity class III (≥ 40 kg/m^2^). To date, the impact of maternal obesity and complex multimorbidity on service provision and healthcare utilisation in NI has not been quantified. Efforts are required to evaluate the ability of services to meet this demand and deliver appropriate care.

### Implications for maternal health care

The high prevalence of multimorbidity in pregnancy alongside health-related risk factors such as advanced maternal age, obesity, and deprivation is likely to have serious implications for women, families and healthcare services in NI. Higher numbers of coexisting conditions impact maternal morbidity and mortality, acute healthcare use in the perinatal period, and places a substantial burden of responsibility on women to coordinate their own care in the perinatal period, often in the absence of evidence informed advice [20–22]. Substantial investment by commissioners and policy makers is required across public health, primary and community care, and maternity services to support women with multimorbidity throughout their reproductive journeys from preconception, during their pregnancies and onwards through long-term postnatal follow-up.

The MBRRACE-UK report (2023) calls for urgent development of maternal medicine specialist centres to facilitate early discussion of complex maternity cases with expert clinicians in order to save lives and improve outcomes for mothers and offspring [16]. As this research has demonstrated, there are a wide range of organ systems involved in multimorbidity in pregnancy, and the intersectional estimates reported here will be useful for designing specialist centres able to provide an array of appropriate services and developing such clinical capacity in NI.

This study confirmed that maternal multimorbidity prevalence followed a social gradient. It follows that efforts are needed in this population to improve social determinants of health. Adequate health care must also be targeted towards communities who are disproportionately affected, and due to differences in how conditions cluster within different groups of women, a variety of services will be necessary. A focus on reducing non-communicable physical conditions such as hypertension and obesity is required for older maternal age groups, particularly in areas that are more deprived. Conversely, greater focus on improving mental health is needed in younger women. However, preconception health and care policies in the UK and Ireland currently lack focus on mental health [23]. It is important that women with multimorbidity are facilitated to engage with services prior to conception to optimise management of their long-term conditions in relation to pregnancy, including advice on medication safety and optimising their nutrition in pregnancy [23]. Women also need equitable access to preconception counselling to discuss how their long-term conditions may be impacted by pregnancy. It therefore follows that interdisciplinary training and education within primary, community and secondary care professionals is required to raise awareness and enact system wide change across all health professionals who care for women with long-term conditions and multimorbidity who have the potential to become pregnant.

### Comparison to other regions

At a population-level, maternal multimorbidity is higher in NI compared to other UK nations. Within the MuM-PreDiCT Collaboration, NI and Scotland’s approaches are broadly similar. Both use a combination of secondary care diagnostic codes and community dispensed medications to ascertain long-term conditions. Data from Lee et al. (2022) presented maternal multimorbidity prevalence in Scotland to be around 19.8%, whereas this study observed a slightly higher rate of 21.7% in NI for the same year (2018) [6]. It cannot be ruled out that there are remaining differences in the healthcare data ecosystems between Scotland and NI which may explain this difference, rather than the health of the two populations.

It is important to note that deprivation levels are higher in NI compared to other regions within the UK, with 37% of the NI population living in the most deprived 20% of the UK, and no areas within NI existing within the most affluent 20% of the UK [7]. Therefore, the patterns in prevalence in of multimorbidity across deprivation quintiles in NI may not be directly comparable to other nations.

### Utility of routinely collected healthcare data – lessons learned

This study highlights the limitations of individual healthcare datasets, and strengthens the case for linkage of maternity data to other types of healthcare data for detection of long-term health conditions, which echoes the findings of MacRae et al. 2023 [15]. For a full discussion, please see Appendix 1 in Supplementary Material.

### Strengths and limitations

The cohort included all pregnancies from all individuals and prevalence was calculated yearly in recognition of several background processes that may impact detection of multimorbidity. Firstly, rates of long-term conditions contributing to multimorbidity may have changed over time in this population. This approach also recognised that the quality of clinical coding practices may have improved over time and that longer look-back periods are available for individuals with pregnancies occurring later in the study period. Stratification of prevalence to different time periods and different maternal characteristics also facilitates comparability of the findings to other contemporaneous cohorts. Retention of all pregnancies and not just index pregnancies in the cohort, also ensured that annual prevalence rates were more accurate.

Stratifying by year also allowed the significant decrease in prevalence of maternal multimorbidity in 2020 to be observed. This drop which was seen across most groups may be explained by the COVID-19 pandemic. This was a period of uncertainty as new evidence was emerging that pregnant women had increased risk, compared to nonpregnant women, of severe illness and death from COVID-19, and yet were not recognised as a high-priority group for vaccination in the early phases of the pandemic [24, 25]. It has been reported that during this time, pregnancy intention rates were generally lower [26] and that there was reduced access to fertility services [27]. Women with pre-existing physical and mental health conditions may have altered their reproductive choices and behaviours resulting in fewer pregnancies to mothers with multimorbidity entering the maternity system, and hence producing the observed drop in prevalence in 2020.

Research projects using administrative health data are bound by statistical disclosure control ensuring that individuals’ information cannot be identified. In NI, results of such projects must not contain a count of less than ten. This has implications for the granularity of data provided to researchers, and for how researchers must present results. For example, ethnicity information was provided to researchers in NI in aggregated groups “white”, “non-white” and “unknown”. This prevents a meaningful interrogation of health disparities between ethnic groups. This study also found a much smaller prevalence of multimorbidity in non-white pregnant women, which may in fact reflect a lower interaction with the health service for newcomers to NI and hence lower detection of conditions, rather than true prevalence. Additionally, this study aggregated age using 10-year intervals (< 25, 25 to 34, and ≥ 35 years) when evaluating prevalence of multimorbidity across intersectional groups. This may mean that prevalence of multimorbidity observed in the extremes of age (< 20 and > 40 years) may have been underestimated.

The approach used in this study to detect long-term conditions from medications required a record of at least two prescriptions in any 12-month period, as opposed to four in the study by Lee et al. 2022 [6]. This was chosen due to the acknowledged reduced scan rates of items into the EPD. The advantage of this approach is increased sensitivity for detection of long-term conditions. However, it should also be highlighted that this could have been at the expense of specificity and may have increased the likelihood of including instances where medications were prescribed for temporary conditions.

### Implications and future research

Ongoing research in NI and more broadly across the UK is exploring the consequences of multimorbidity in pregnant women on outcomes in the antenatal, peripartum and postnatal period for both mother and infant [28, 29]. These studies will explore the impact of the number of coexisting conditions, how particular combinations of physical and mental health conditions impact outcomes, and which combinations of conditions confer the highest risk. This study highlights the importance of detailed evaluation of subtypes of multimorbidity. In particular, our results demonstrate divergent patterns in prevalence of mental health and physical multimorbidity across different groups of pregnant women.

Methodological insights from the sub analysis in Appendix 1 have important implications for research utilising routinely collected administrative health data. This is particularly relevant with regard operationalising multimorbidity phenome definitions within different types of routinely collected healthcare data, for example from secondary care diagnoses, medication dispensations and self-reported past medical histories which, as demonstrated by this study, can provide different estimates for prevalence of long-term conditions.

This study also highlights important, unanswered research questions regarding inequalities in the burden of multimorbidity across intersectional groups of women in NI. In particular, there is a need to understand the social and economic determinants of the higher burden of mental health morbidity in the more deprived and younger pregnant women, and the consequences of this higher burden for maternal and child outcomes. Potential factors pertinent to NI have been posited by others including prevalence of violence against women and girls, intergenerational legacy of “the troubles”, and the impact of austerity [30, 31]. There may also be an increased awareness and willingness to seek help amongst the younger age groups. There has also been a suggestion that health professionals’ decision making can be influenced by implicit bias which can exacerbate health inequalities in individuals with low socioeconomic status [32].

## Conclusion

This study used a robust analytic approach in line with previous MuM-PreDICT epidemiological studies of pre-existing multimorbidity in pregnant women. It has shown that multimorbidity impacts over 1 in 5 pregnant women living in NI, with complex multimorbidity affecting over 1 in 20 pregnant women in NI, and that differences exist in the prevalence and patterns of physical and mental health conditions across demographic and socioeconomic groups. The varied and distinct combinations of health conditions across intersectional groups point to a need for regional service development to cope with complexity in pregnancy and an individualised approach to provision of preconception health promotion, maternity care and long-term postnatal follow-up.

## Supplementary Information

Supplementary Material 1.

Supplementary Material 2.

Supplementary Material 3.

## Data Availability

The data that support the findings of this study are available from HSCNI HBS but restrictions apply to the availability of these data, which were used under license for the current study, and so are not publicly available.

## Abbreviations

BMI: Body mass index
BNF: British National Formulary
CMHD: Common mental health disorder
EPD: Enhanced Prescribing Database
GP: General Practitioner
HBS: Health and Social Care Northern Ireland Business Service Organisation Honest Broker Service
HCN: Health and Care Number
ICD-10: International Classification of Diseases 10th Revision
MBRRACE-UK: Mothers and Babies: Reducing Risk through Audits and Confidential Enquiries across the UK
NI: Northern Ireland
NIMATS: Northern Ireland Regional Maternity Service Database
NIMDM: NI Multiple Deprivation Measure
PAS: Patient Administration System
UK: United Kingdom
