# Supplementary material for: Prevalence and patterns of pre-existing multimorbidity in pregnancy in Northern Ireland: a population-based, retrospective study using linked routinely collected healthcare data
Source: BMC Pregnancy Childbirth. 2025 Jun 7;25:666. doi: 10.1186/s12884-025-07771-1 (PMC12145590; doi:10.1186/s12884-025-07771-1)
Supplement: Supplementary file 1 — Supplementary Material 1. [file 12884_2025_7771_MOESM1_ESM.docx]

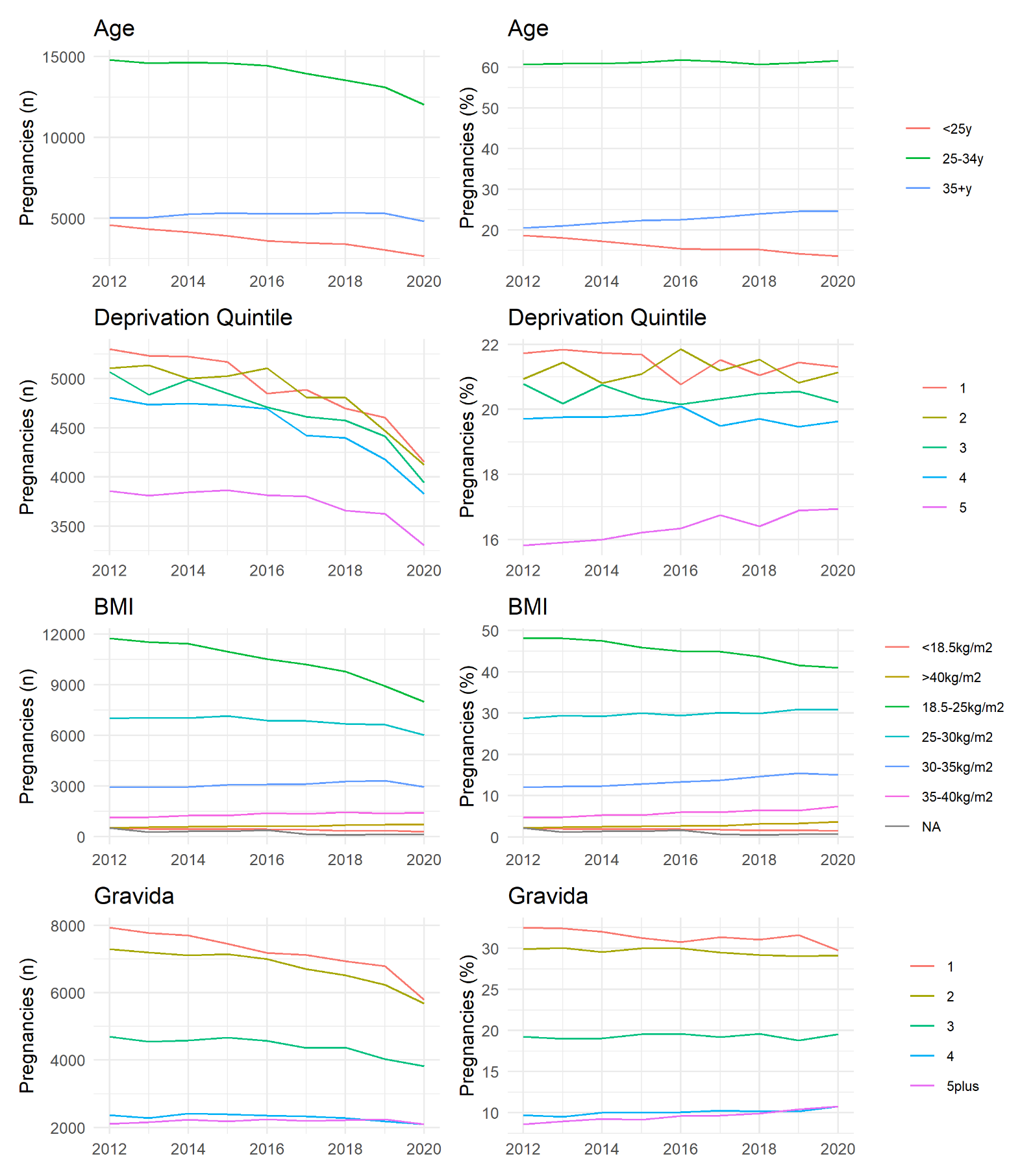


Fig S1: Temporal trends (2012 to 2020) in age, deprivation, BMI and gravida (full cohort)


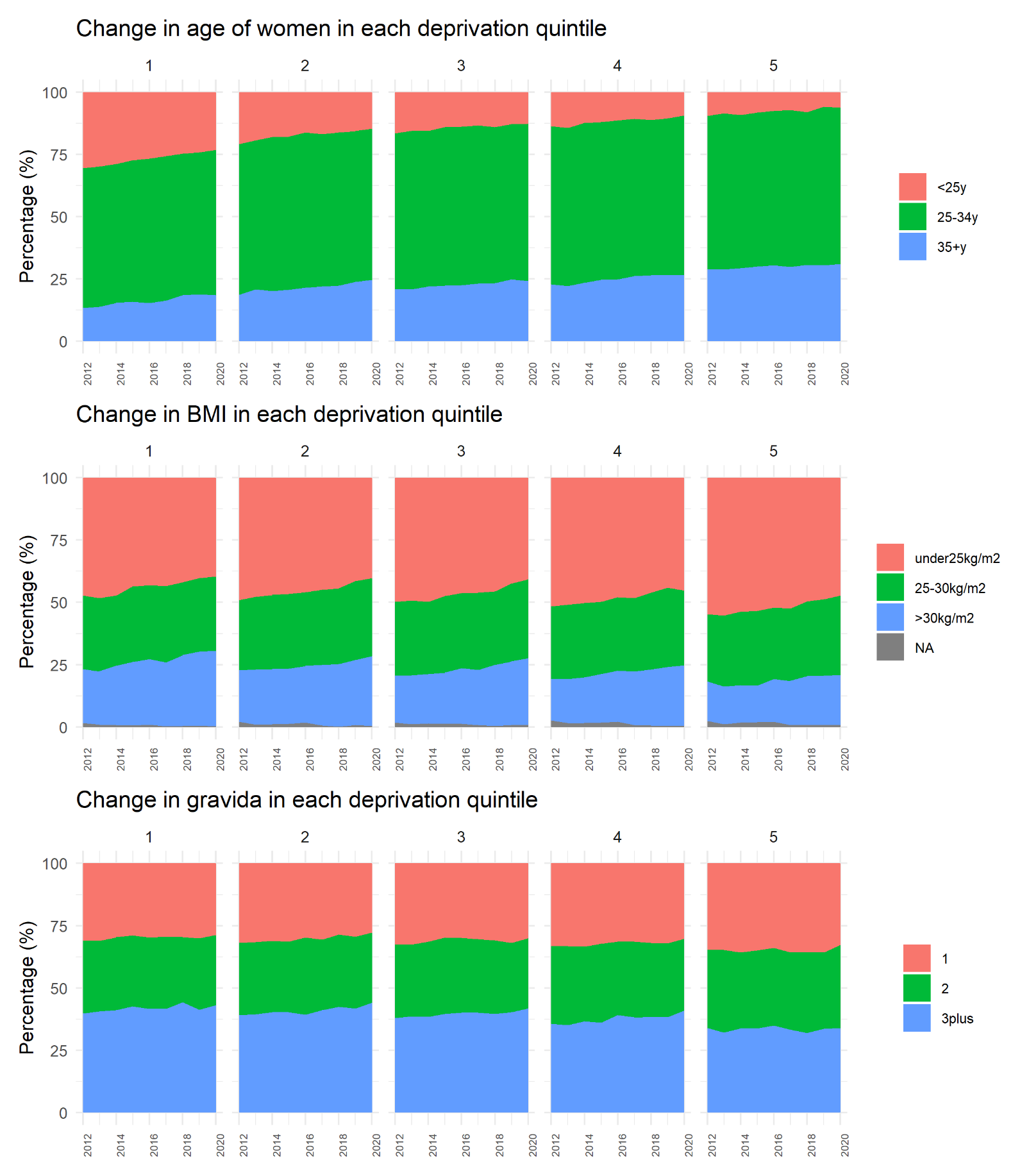


Fig S2: Temporal trends (2012 to 2020) in age, BMI and gravida within each deprivation quintile (1=most deprived) (full cohort)


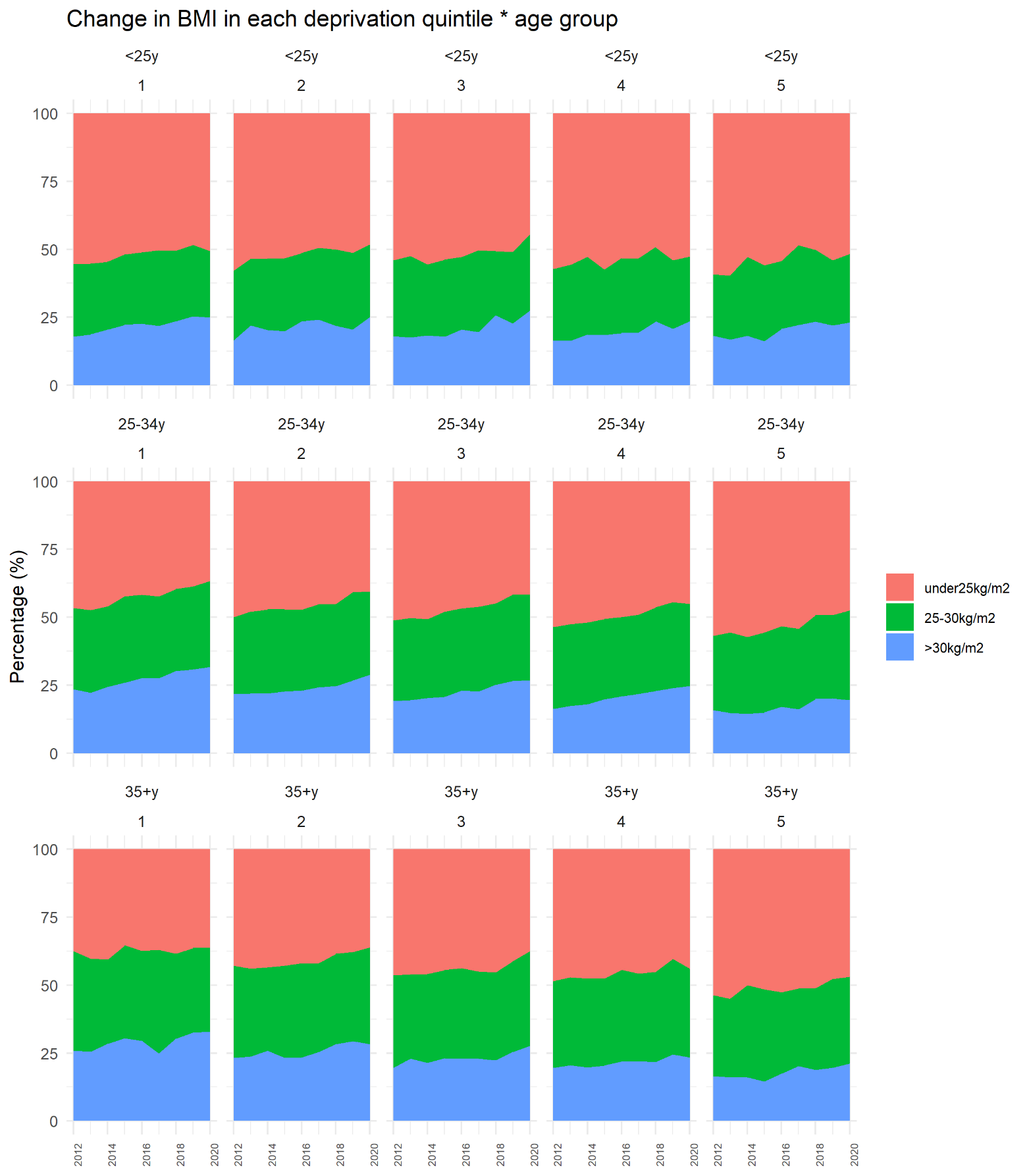


Fig S3: Temporal changes (2012 to 2020) in BMI in each age/deprivation group (1=most deprived)


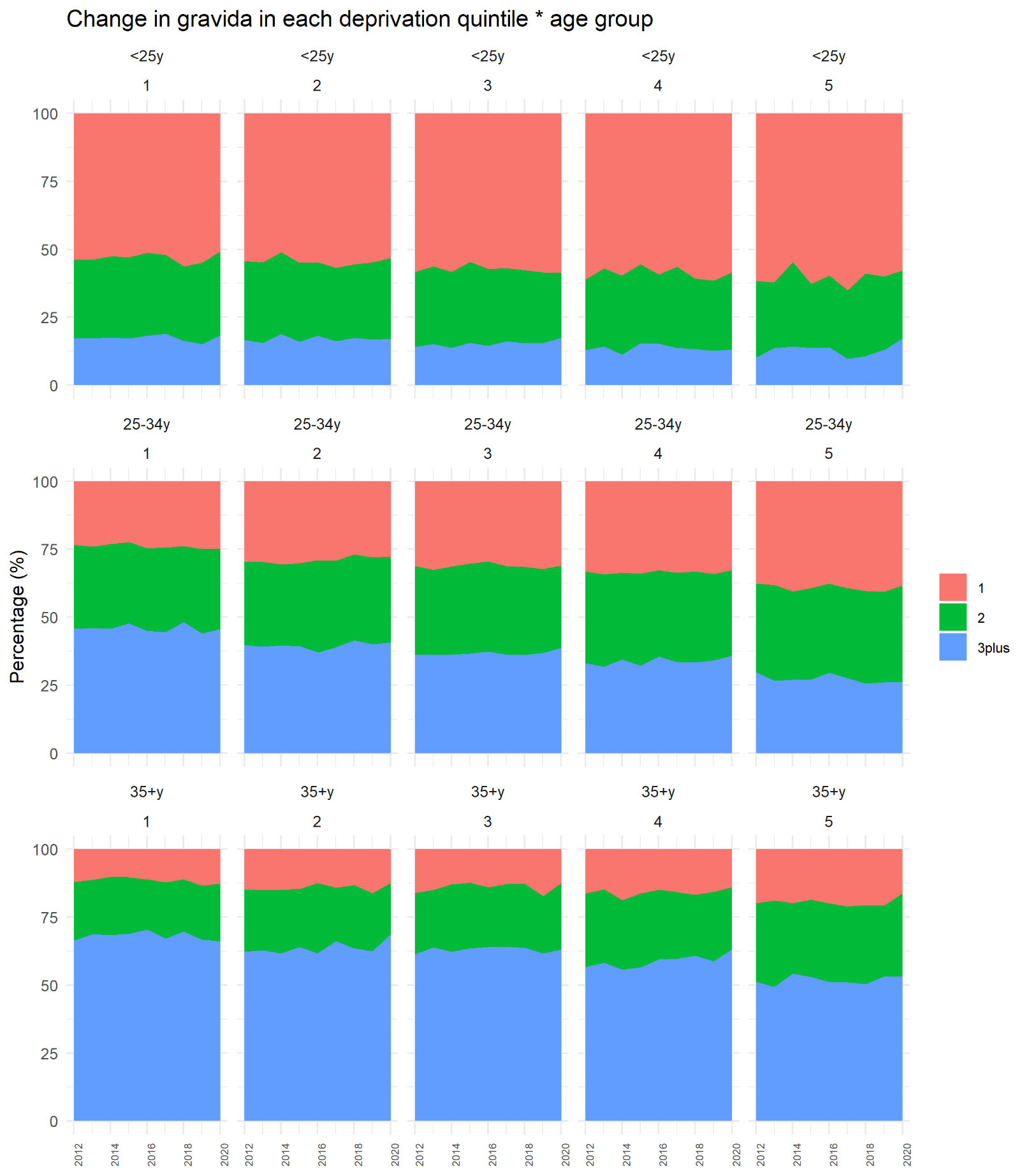


Fig S4: Temporal changes (2012 to 2020) in gravida in each age/deprivation group


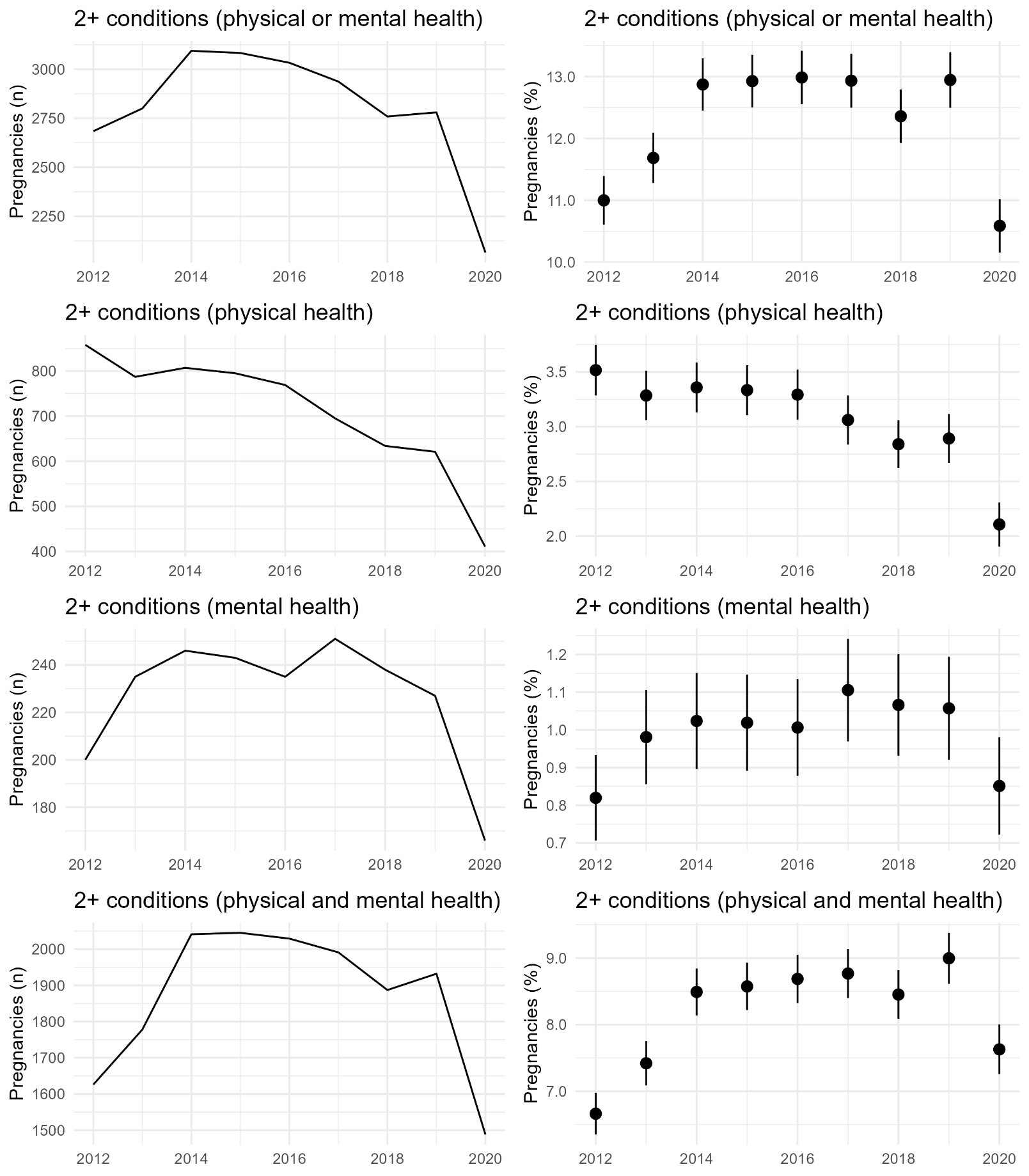


Fig S5: Temporal changes (2012 to 2020) in detectable multimorbidity using a standardised look-back period


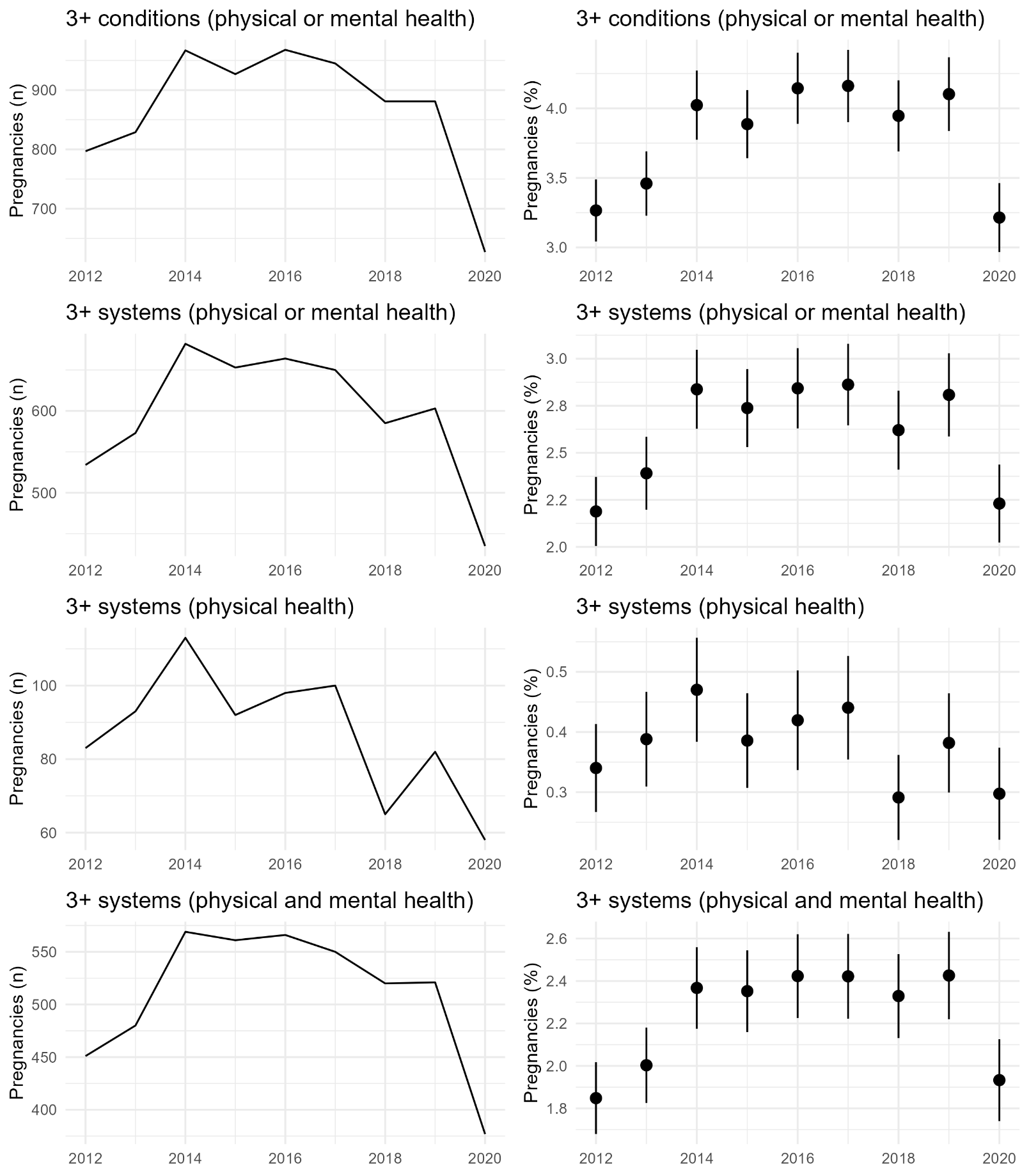


Fig S6: Temporal changes (2012 to 2020) in detectable complex multimorbidity using a standardised look-back period


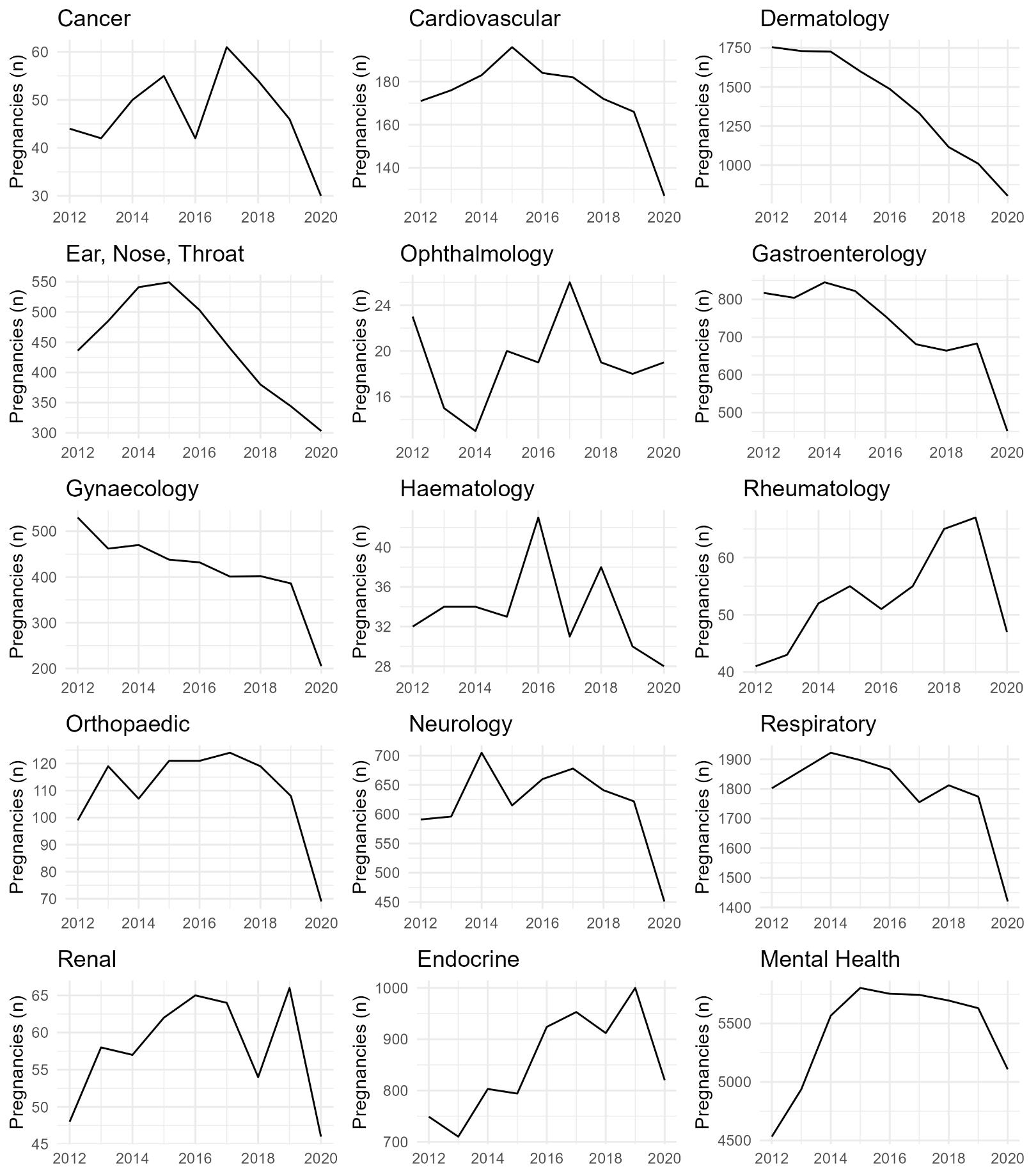


Fig S7: Temporal changes (2012 to 2020) in frequency pregnant women with at least one condition affecting each organ system (detected using a standardised look-back period)


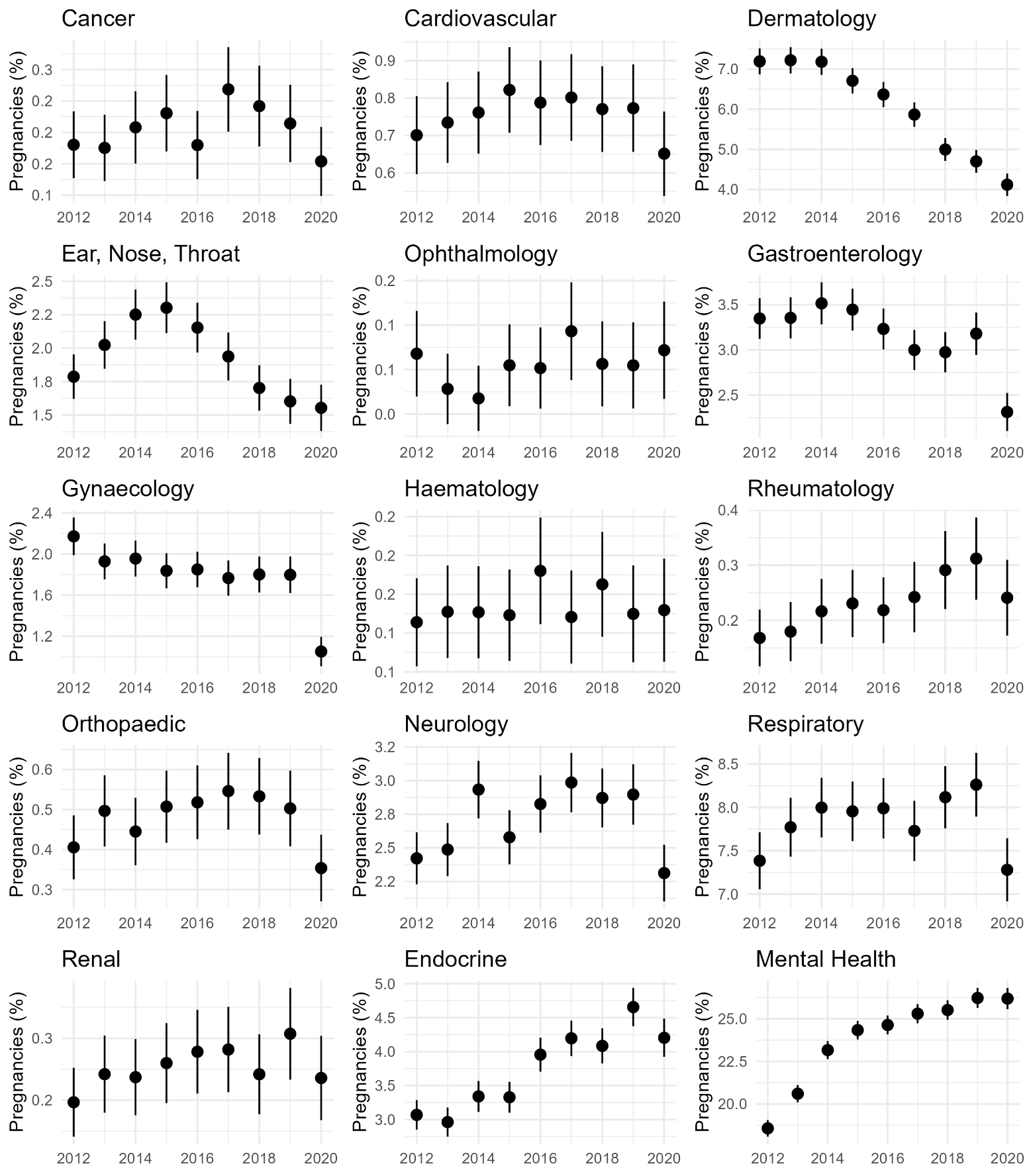


Fig S8: Temporal changes (2012 to 2020) in proportion of pregnant women with at least one condition affecting each organ system (detected using a standardised look-back period)


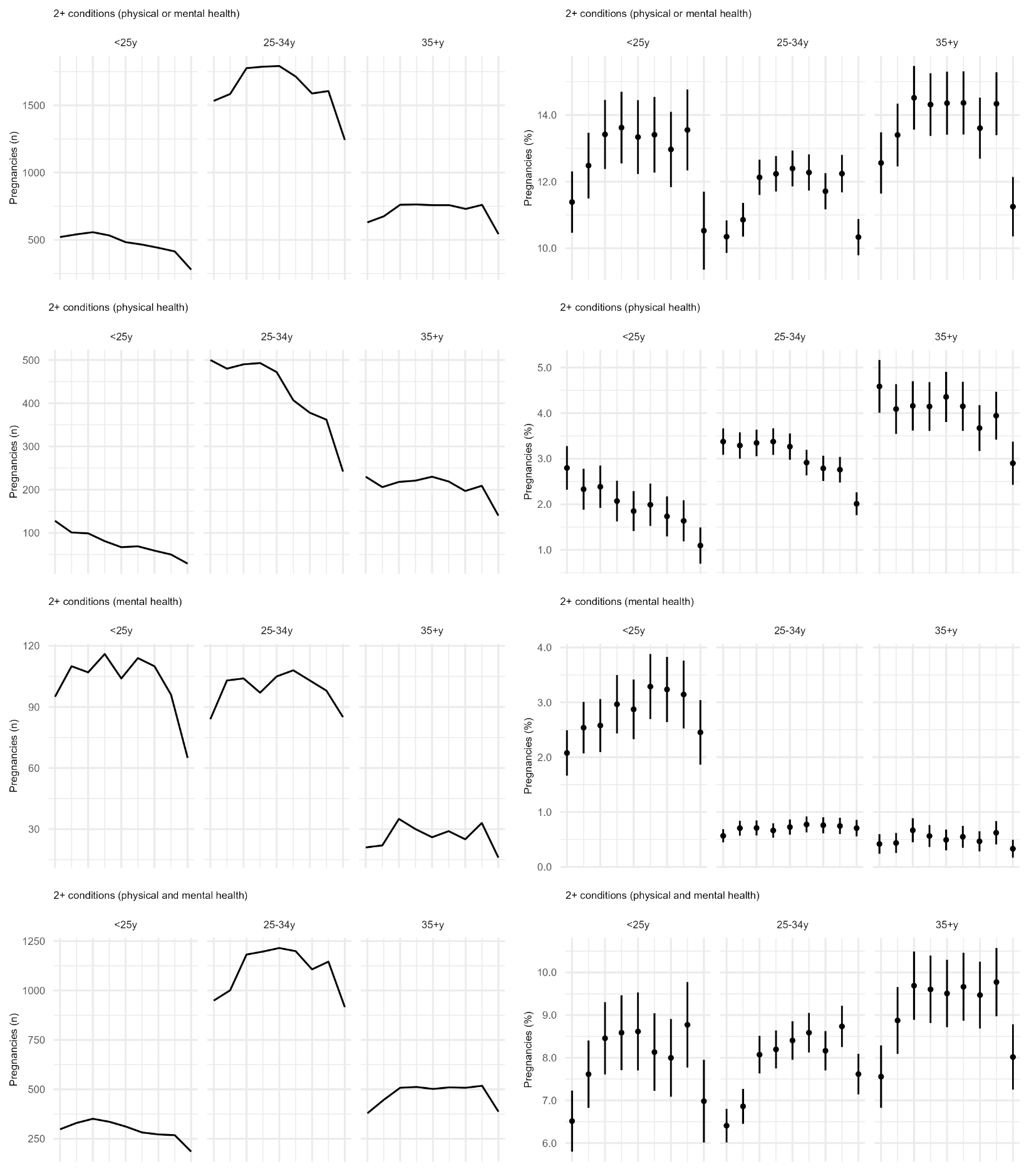


Fig S9: Temporal changes (2012 to 2020) in detectable multimorbidity across age groups using a standardised look-back period


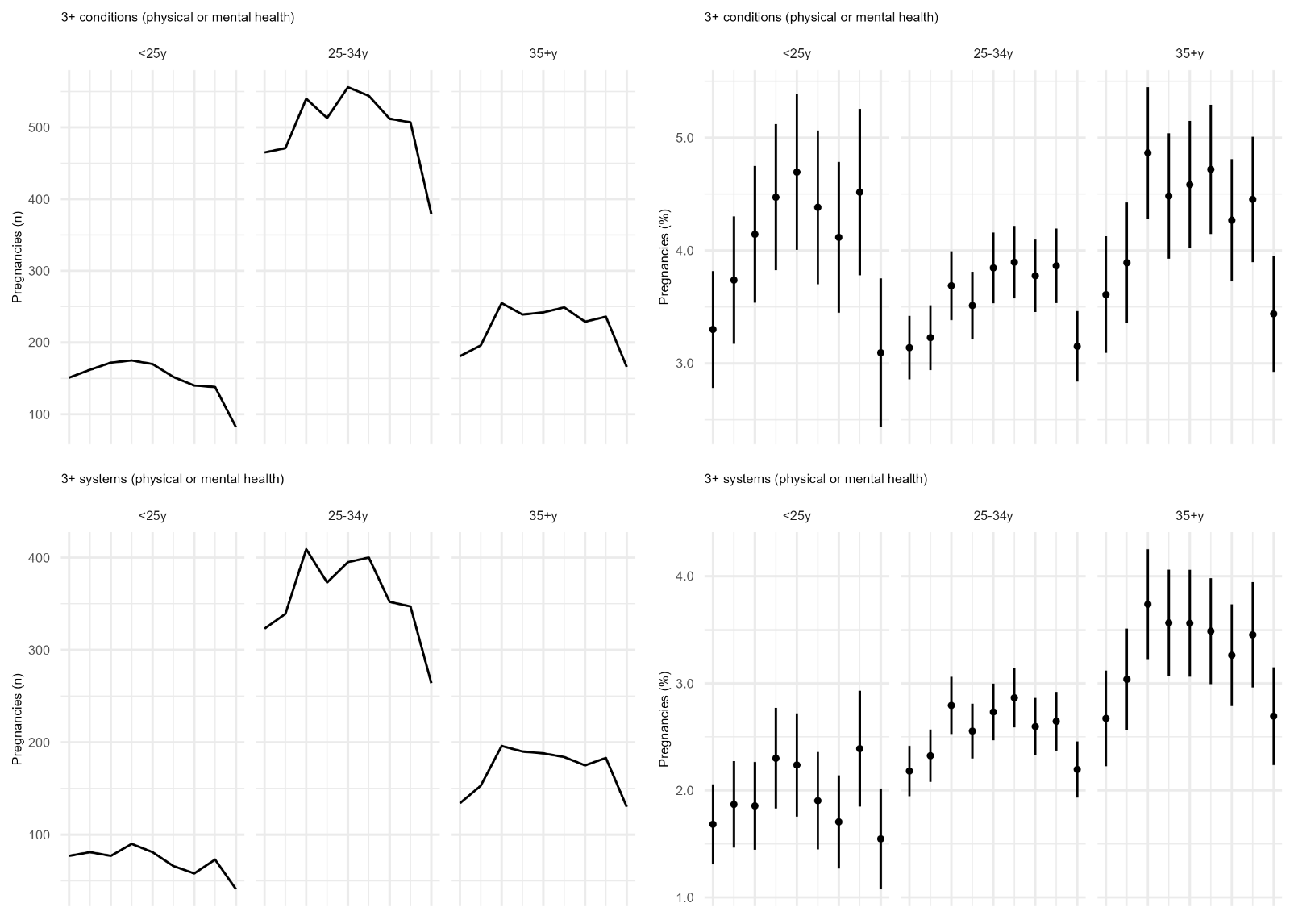


Fig S10: Temporal changes (2012 to 2020) in detectable complex multimorbidity across age groups using a standardised look-back period


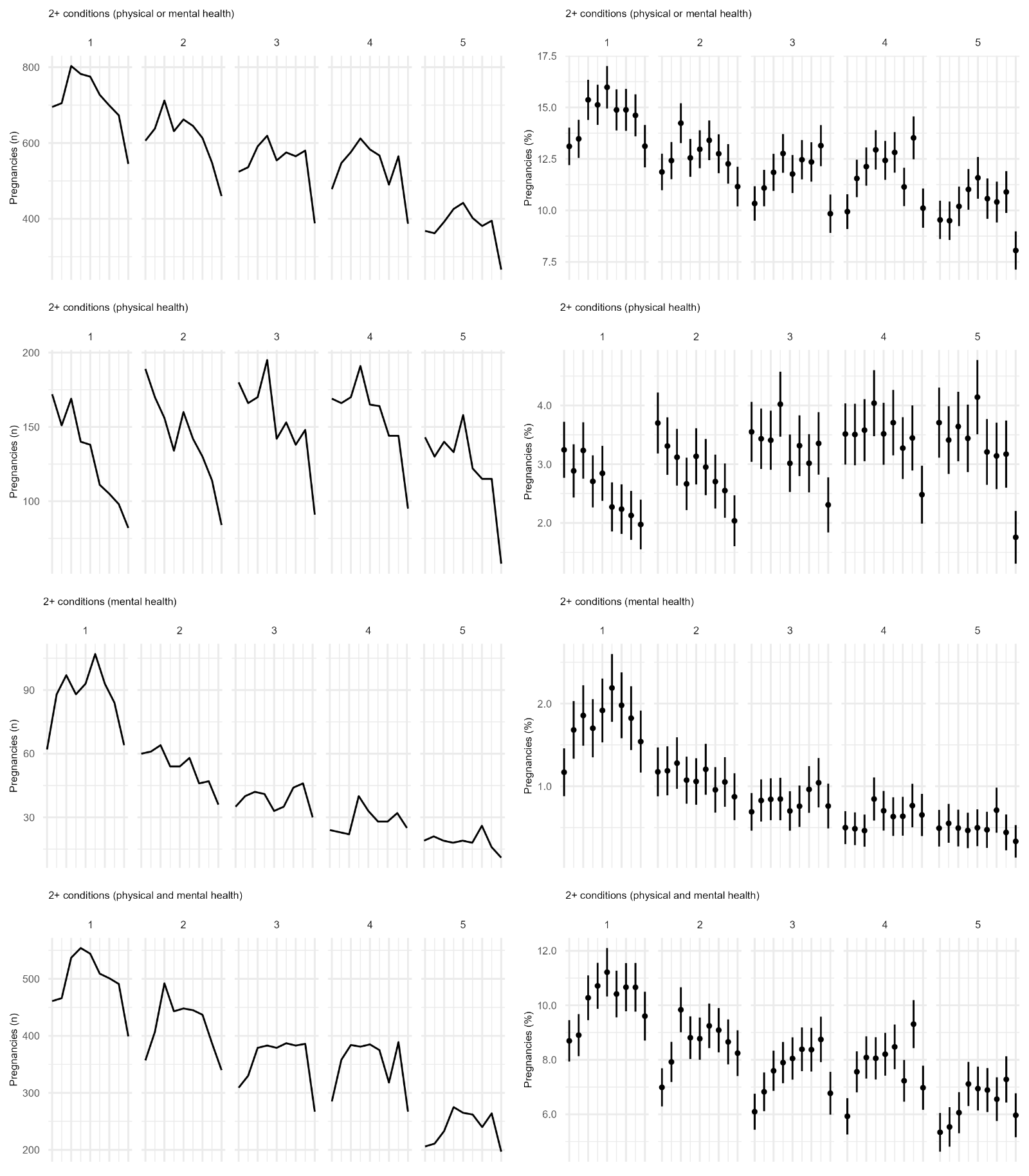


Fig S11: Temporal changes (2012 to 2020) in detectable multimorbidity across deprivation quintiles (1=most deprived) using a standardised look-back period


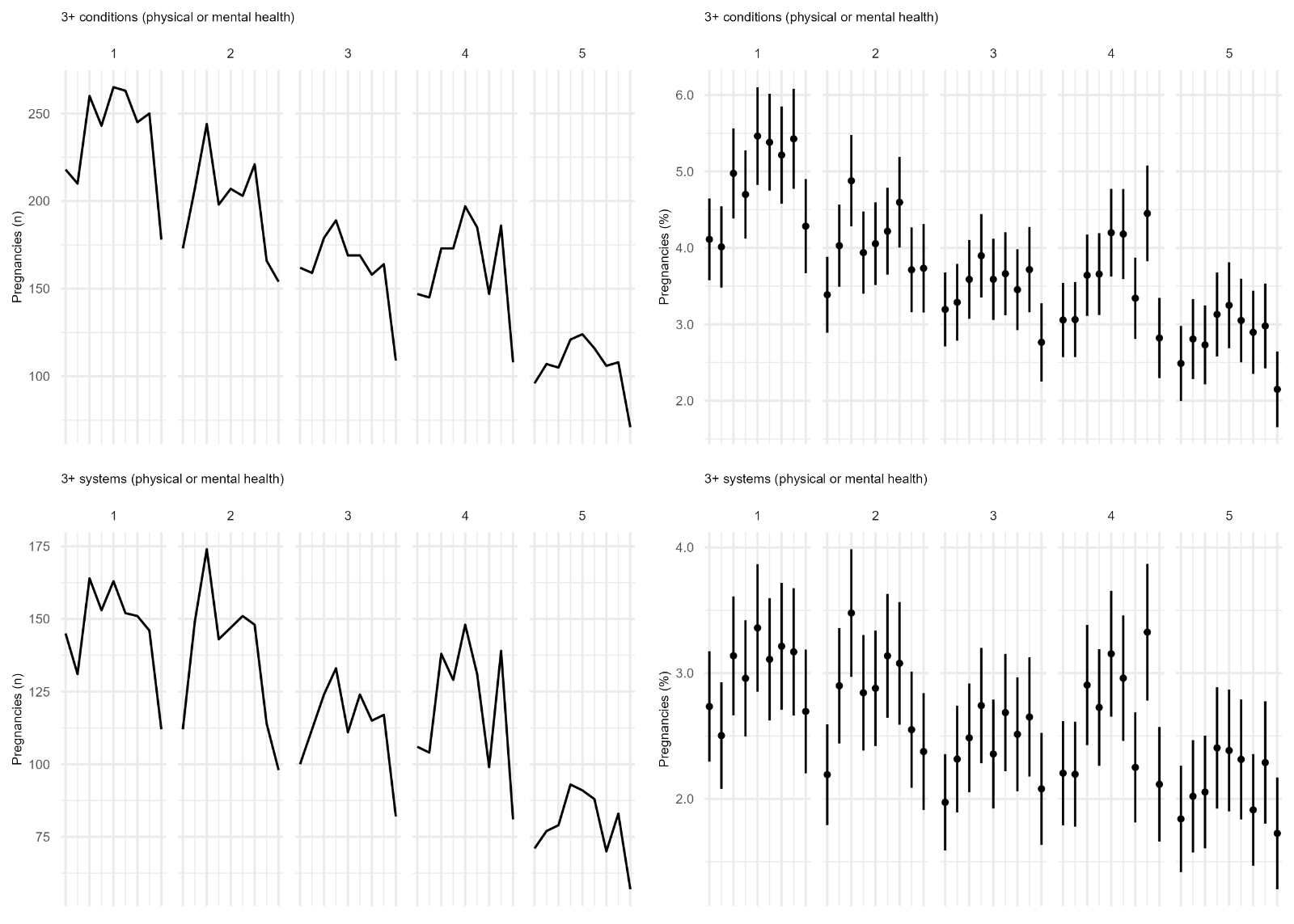


Fig S12: Temporal changes (2012 to 2020) in detectable complex multimorbidity across deprivation quintiles (1=most deprived) using a standardised look-back period


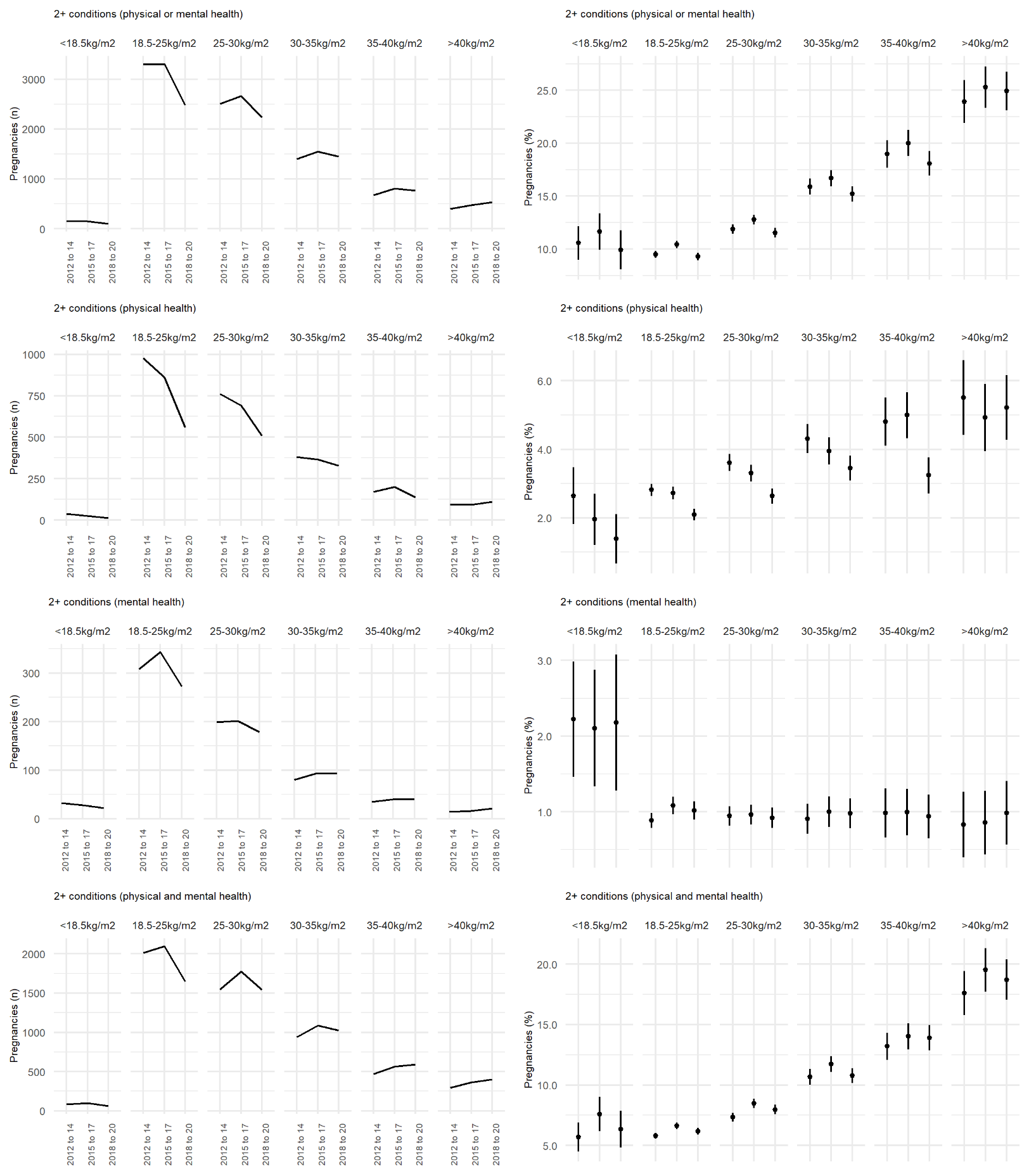


Fig S13: Temporal changes (2012 to 2020) in detectable multimorbidity across BMI groups using a standardised look-back period


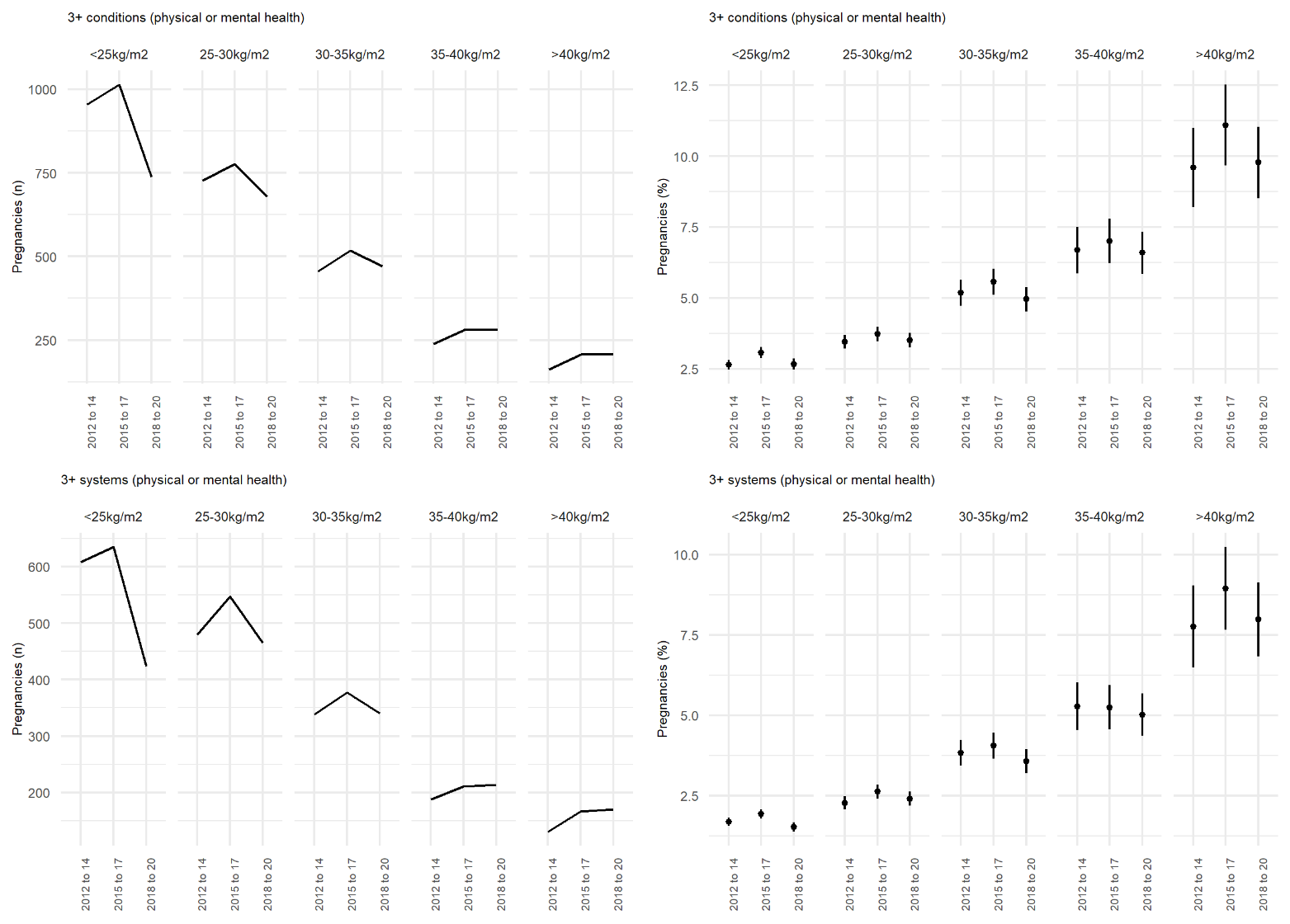


Fig S14: Temporal changes (2012 to 2020) in detectable complex multimorbidity across BMI groups using a standardised look-back period


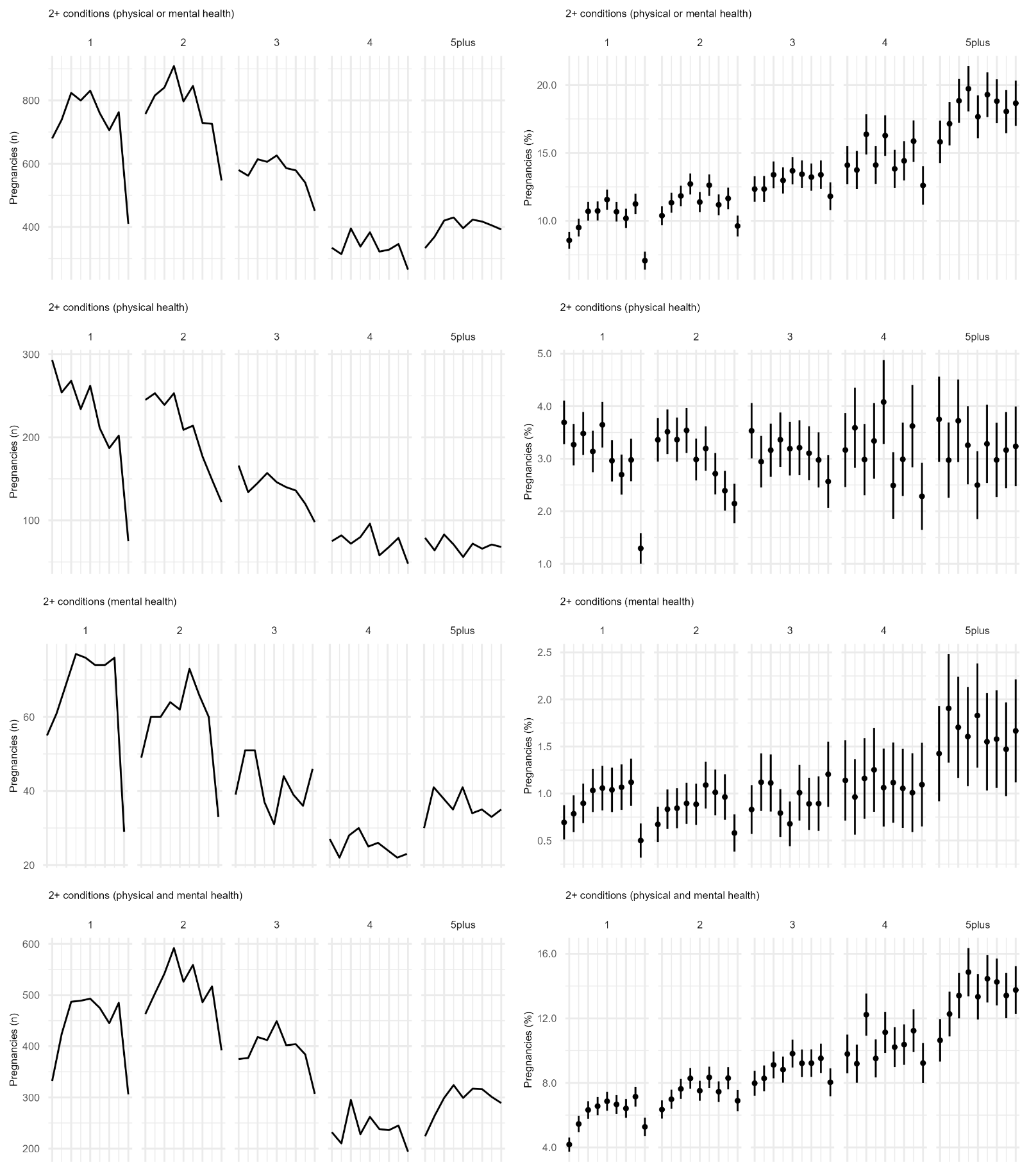


Fig S15: Temporal changes (2012 to 2020) in detectable multimorbidity across gravida groups using a standardised look-back period


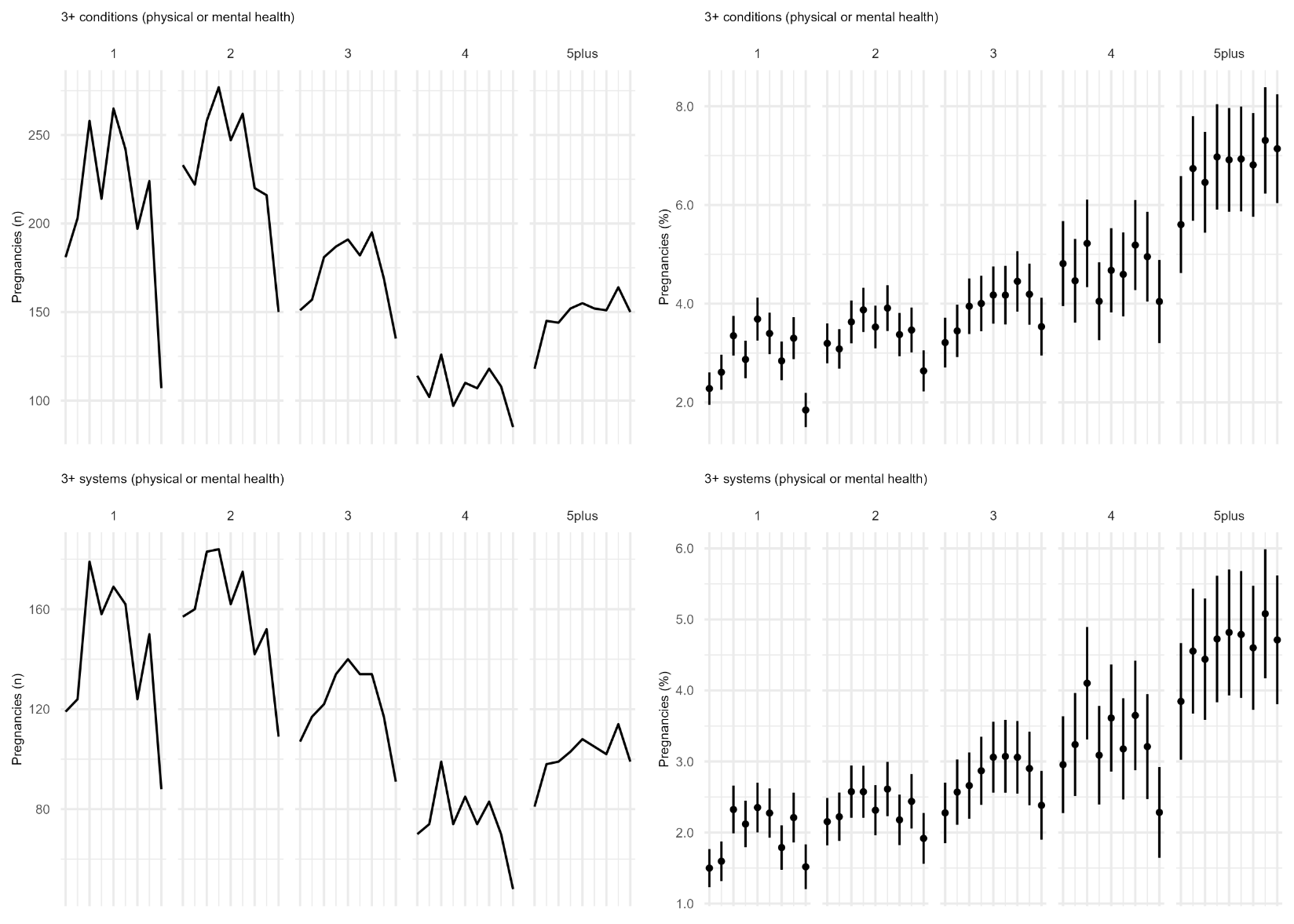


Fig S16: Temporal changes (2012 to 2020) in detectable complex multimorbidity across gravida groups using a standardised look-back period


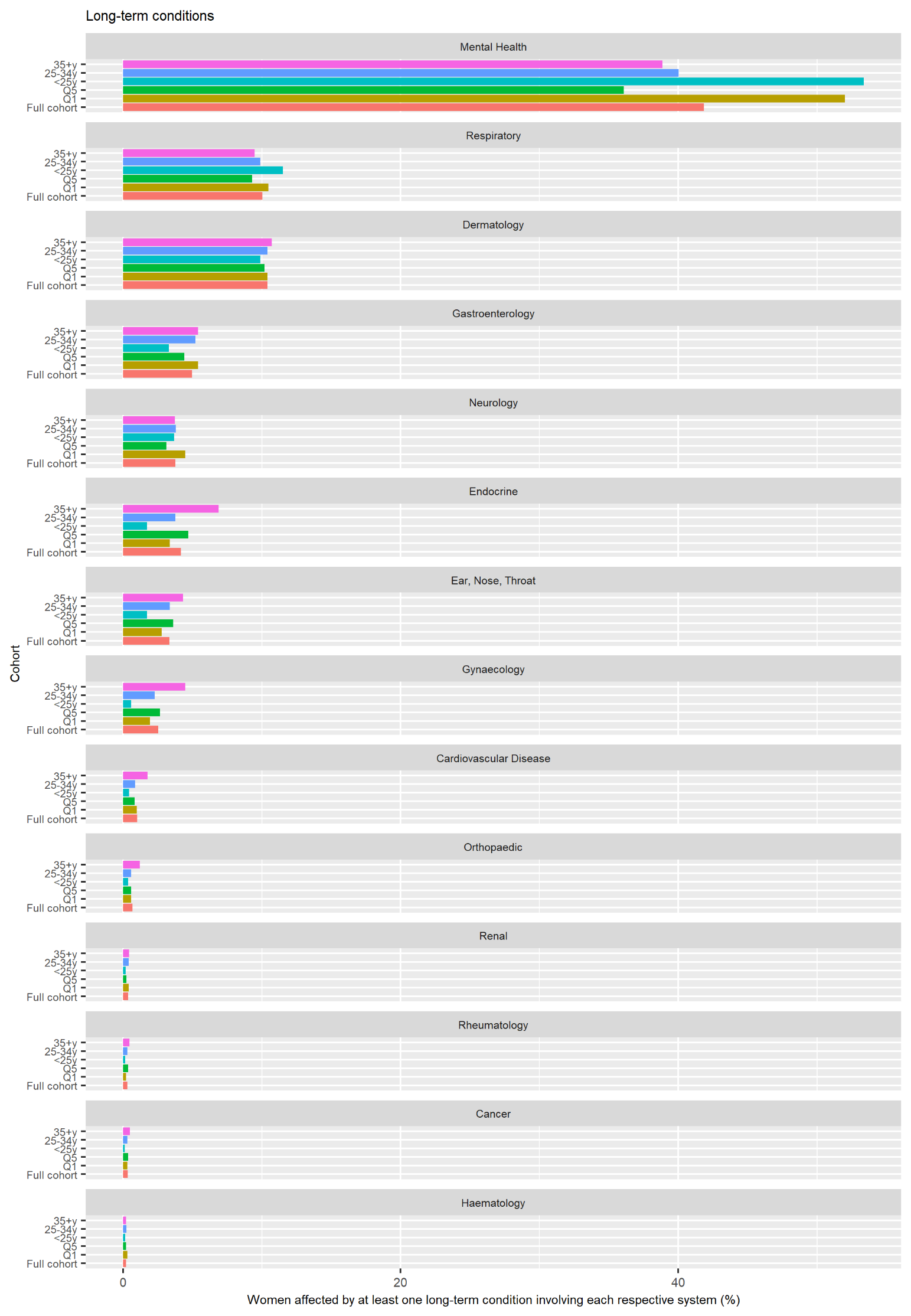


Fig S17: Proportion of pregnant women (2014 to 2019) with at least one condition affecting each organ system (detected using full look-back period)
