# Supplementary material for: Prevalence and patterns of pre-existing multimorbidity in pregnancy in Northern Ireland: a population-based, retrospective study using linked routinely collected healthcare data
Source: BMC Pregnancy Childbirth. 2025 Jun 7;25:666. doi: 10.1186/s12884-025-07771-1 (PMC12145590; doi:10.1186/s12884-025-07771-1)
Supplement: Supplementary file 2 — Supplementary Material 2. [file 12884_2025_7771_MOESM2_ESM.docx]

| **Table S1: Top 10 unique combinations of multimorbidity (2+ conditions) in women in NI with pregnancy start date between 2014 and 2019 (n=137,750)** | | | |
| --- | --- | --- | --- |
| **Physical or mental health** | | | |
| **Rank** |  | **n** | **%** |
| 1 | CMHD + asthma | 3099 | 2.25 |
| 2 | CMHD + eczema | 2877 | 2.09 |
| 3 | CMHD + thyroid disorder | 1040 | 0.75 |
| 4 | CMHD + Other MH | 885 | 0.64 |
| 5 | CMHD + migraine | 838 | 0.61 |
| 6 | CMHD + IBD | 838 | 0.61 |
| 7 | CMHD + allergic rhinoconjunctivitis | 777 | 0.56 |
| 8 | CMHD + asthma + eczema | 641 | 0.47 |
| 9 | Asthma + eczema | 599 | 0.43 |
| 10 | CMHD + gall stones | 464 | 0.34 |
| **Physical health only (2+ conditions)** | | | |
| **Rank** |  | **n** | **%** |
| 1 | Asthma + eczema | 1403 | 1.02 |
| 2 | Asthma + allergic rhinoconjunctivitis | 718 | 0.52 |
| 3 | Eczema + psoriasis | 664 | 0.48 |
| 4 | Eczema + allergic rhinoconjunctivitis | 379 | 0.28 |
| 5 | Eczema + thyroid disorder | 316 | 0.23 |
| 6 | Asthma + IBD | 301 | 0.22 |
| 7 | Asthma + thyroid disorder | 288 | 0.21 |
| 8 | Asthma + migraine | 265 | 0.19 |
| 9 | Eczema + IBD | 257 | 0.19 |
| 10 | Eczema + migraine | 218 | 0.16 |

*CMHD = Common Mental Health Disorder; Other MH = Other Mental Health Condition; IBD = Irritable Bowel Disease*

| **Table S2: Top 10 unique combinations of systems involved in complex multimorbidity (3+ conditions from 3+ systems) in women in NI with pregnancy start date between 2014 and 2019 (n=137,750)** | | | |
| --- | --- | --- | --- |
| **Rank** | **Complex Multimorbidity**  **Physical or mental health (3+ systems)** | **n** | **%** |
| 1 | Mental health + Respiratory + Dermatology | 912 | 0.66 |
| 2 | Mental health + Respiratory + GI | 421 | 0.31 |
| 3 | Mental health + Respiratory + ENT | 375 | 0.27 |
| 4 | Mental health + Dermatology + GI | 357 | 0.26 |
| 5 | Mental health + Respiratory + Neurology | 350 | 0.25 |
| 6 | Mental health + Dermatology + Neurology | 254 | 0.18 |
| 7 | Mental health + Dermatology + Endocrine | 223 | 0.16 |
| 8 | Mental health + Respiratory + Endocrine | 208 | 0.15 |
| 9 | Mental health + Dermatology + ENT | 206 | 0.15 |
| 10 | Mental health + Neurology + GI | 189 | 0.14 |

GI = Gastroenterology; ENT = Ear, Nose, Throat

| **Table S3: Top 10 unique combinations of multimorbidity (2+ conditions) in women in NI with pregnancy start date between 2014 and 2019, stratified by age** | | | | | | | | | |
| --- | --- | --- | --- | --- | --- | --- | --- | --- | --- |
|  | **Age <25y**  **n=21,609** | | | **Age 25 – 34y**  **n=84,346** | | | **Age 35+y**  **n=31,795** | | |
| **Rank** | **Conditions** | **n** | **%** | **Conditions** | **n** | **%** | **Conditions** | **n** | **%** |
| 1 | CMHD + Asthma | 250 | 3.22 | CMHD + Asthma | 443 | 2.64 | CMHD + Eczema | 626 | 1.97 |
| 2 | CMHD + Eczema | 191 | 2.46 | CMHD + Eczema | 383 | 2.28 | CMHD + Asthma | 559 | 1.76 |
| 3 | CMHD + Other MH | 146 | 1.88 | CMHD + Other MH | 149 | 0.89 | CMHD + Thyroid disorder | 405 | 1.27 |
| 4 | CMHD + Migraine | 67 | 0.86 | CMHD + Migraine | 125 | 0.75 | CMHD + Allergic Rhinoconjunctivitis | 237 | 0.75 |
| 5 | CMHD + Eczema | 63 | 0.81 | CMHD + IBD | 117 | 0.7 | CMHD + IBD | 162 | 0.51 |
| 6 | SMI + Other MH | 58 | 0.75 | CMHD + thyroid disorder | 104 | 0.62 | CMHD + Migraine | 152 | 0.48 |
| 7 | CMHD + IBD | 46 | 0.59 | CMHD + gall stones | 92 | 0.55 | CMHD + Asthma + Eczema | 120 | 0.38 |
| 8 | Depression* + Other MH | 37 | 0.48 | CMHD + Allergic Rhinoconjunctivitis | 84 | 0.5 | Asthma + Eczema | 119 | 0.37 |
| 9 | SMI + Asthma | 33 | 0.43 | CMHD + Asthma + Eczema | 83 | 0.49 | CMHD + Gall stones | 117 | 0.37 |
| 10 | CMHD + Neurodevelopmental disorder | 29 | 0.37 | SMI + Asthma | 65 | 0.39 | CMHD + Infertility | 109 | 0.34 |

*CMHD = Common Mental Health Disorder; SMI = Serious Mental Health Disorder; Other MH = Other Mental Health Condition; IBD = Irritable Bowel Disease*

**Depression identified from ICD10 code diagnosis (associated with a secondary care admission)*

| **Table S4: Top 10 unique combinations of multimorbidity (2+ conditions) in women in NI with pregnancy start date between 2014 and 2019, stratified by deprivation** | | | | | | |
| --- | --- | --- | --- | --- | --- | --- |
|  | **Most deprived**  **N=29,439** | | | **Least deprived**  **N=22,617** | | |
| **Rank** | **Conditions** | **N** | **%** | **Conditions** | **N** | **%** |
| 1 | CMHD + Asthma | 797 | 2.71 | CMHD + Eczema | 454 | 2.01 |
| 2 | CMHD + Eczema | 692 | 2.35 | CMHD + Asthma | 428 | 1.89 |
| 3 | CMHD + Other MH | 310 | 1.05 | CMHD + Thyroid disorder | 210 | 0.93 |
| 4 | CMHD + Migraine | 217 | 0.74 | CMHD + Allergic Rhinoconjunctivitis | 141 | 0.62 |
| 5 | CMHD + IBD | 195 | 0.66 | CMHD + IBD | 136 | 0.6 |
| 6 | CMHD + Thyroid disorder | 183 | 0.62 | CMHD + Migraine | 131 | 0.58 |
| 7 | CMHD + Asthma + Eczema | 162 | 0.55 | Asthma + Eczema | 107 | 0.47 |
| 8 | CMHD + Allergic Rhinoconjunctivitis | 141 | 0.48 | CMHD + Asthma + Eczema | 96 | 0.42 |
| 9 | CMHD + Gall stones | 127 | 0.43 | Asthma + Allergic Rhinoconjunctivitis | 83 | 0.37 |
| 10 | SMI + Asthma | 112 | 0.38 | CMHD + Other MH | 81 | 0.36 |

*CMHD = Common Mental Health Disorder; SMI = Serious Mental Illness; Other MH = Other Mental Health Condition; IBD = Irritable Bowel Disease*
