# Supplementary material for: Prevalence and patterns of pre-existing multimorbidity in pregnancy in Northern Ireland: a population-based, retrospective study using linked routinely collected healthcare data
Source: BMC Pregnancy Childbirth. 2025 Jun 7;25:666. doi: 10.1186/s12884-025-07771-1 (PMC12145590; doi:10.1186/s12884-025-07771-1)
Supplement: Supplementary file 3 — Supplementary Material 3. [file 12884_2025_7771_MOESM3_ESM.docx]

**APPENDIX 1**

**Sub-analysis: utility of different data sources for detecting conditions**

**Aim:** To explore the utility of NI’s healthcare datasets for detecting health conditions and multimorbidity over time (2012-2020, inclusive).

**Methods:** Further analysis of the utility of three different types of routinely collected data for detection of one physical condition (asthma) and one mental health condition (common mental health disorder (CMHD), defined as either anxiety or depression) was conducted. Detection rates for each condition were compared on a yearly basis using (1) secondary care diagnostic codes (ICD-10), (2) GP prescribed community dispensed medications and (3) past medical history variables obtained from NIMATS. This sub-analysis is included as an illustration for researchers using routinely collected data to analyse specific health conditions.

**Results:** The three databases resulted in vastly different magnitudes of prevalence in our chosen example conditions of asthma and CMHD (**Fig 1)**. Using medications only, asthma was estimated to impact between 7.2-9.3% pregnancies, whereas secondary care diagnosis codes estimated a much lower prevalence of pre-existing asthma (2.0-4.4%). Combining both medications and diagnostic codes estimated the prevalence of pre-existing asthma to be between 7.8 and 10.5%. Past medical history variables in NIMATS estimated a much higher prevalence of pre-existing asthma, ranging between 15.1 and 16.8%.

For CMHD, medications estimated prevalence to be 14.9% in 2012 increasing to 23% in 2020, whereas secondary care diagnostic codes detected pre-existing CMHD in only 1.2 to 3.0% of pregnancies. Information from NIMATS showed discrepant rates compared to medication-identified CMHD, being lower in 2012 at 12.0% of pregnancies, but higher in 2020 at 31% of pregnancies.


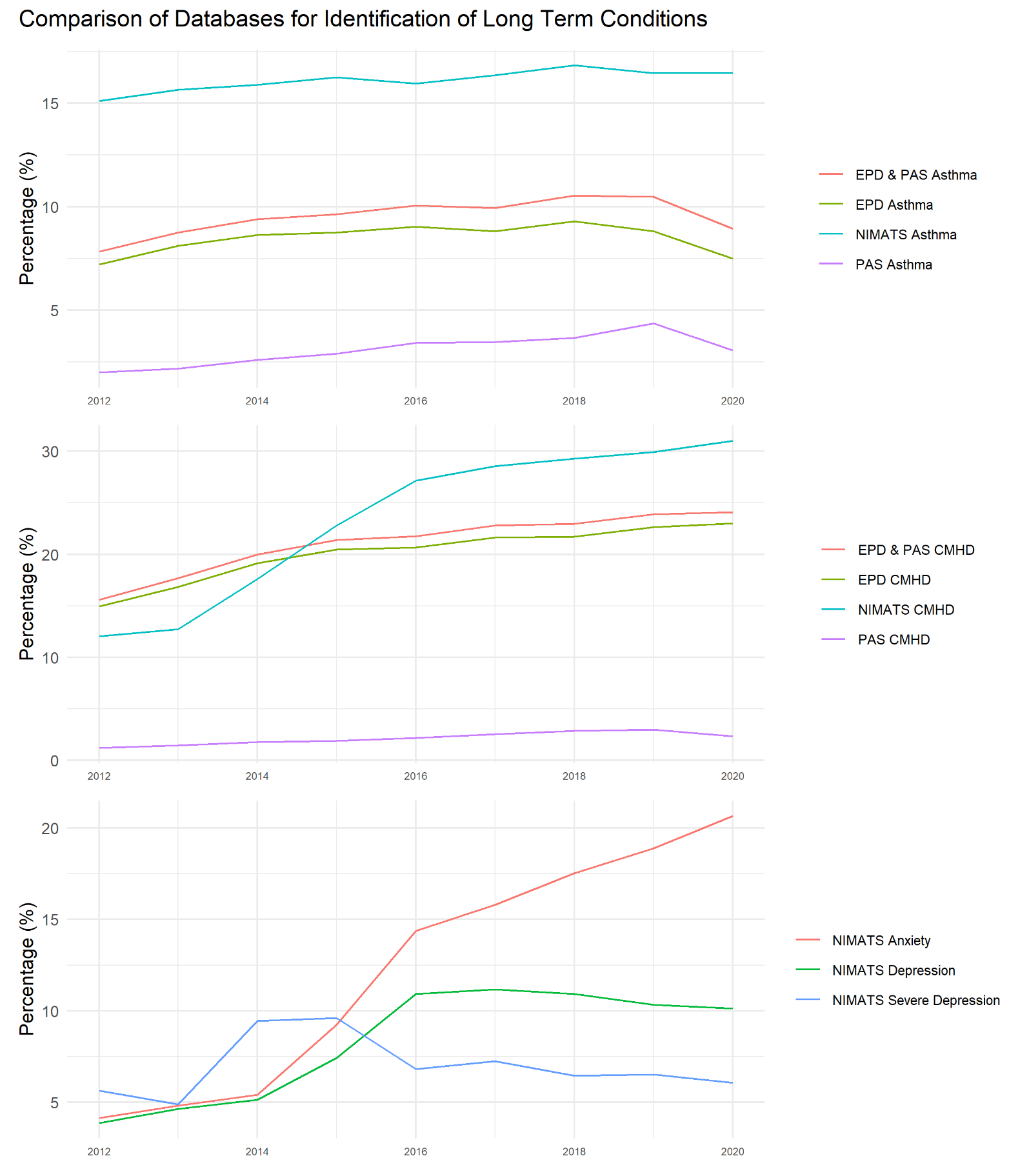


Fig 1: Temporal changes (2012 to 2020) in proportion of pregnant women detected within each database with at least one condition (detected using a full look back period for comparability with NIMATS)

*EPD = Enhanced Prescribing Database (BNF codes); PAS = Patient Administration System (ICD-10 codes); NIMATS = Northern Ireland Maternity System (self-reported or manually transcribed from GP letters)*

**Discussion:** This study highlights the limitations of individual healthcare datasets, and strengthens the case for linkage of maternity data to other types of healthcare data for detection of long-term health conditions, which echoes the findings of MacRae et al 2023.[1] Our sub-analysis comparing detection rates from NIMATS (maternity records), EPD (community dispensed medications) and PAS (hospital admission diagnoses) for two common conditions, asthma and common mental health disorder, demonstrated that prevalence rates can be vastly different between databases, with each also varying over time. The design and purpose of databases should be borne in mind when utilising each to detect long-term conditions.

NIMATS past medical history variables are manually recorded at the time of booking, and rely on a combination of self-report from women and details within GP referral letters. Variation in data collection, added to variations over time in the design of this clinical system can impact its usefulness for surveillance of long-term conditions in pregnancy.

Specific to the EPD database, previous research demonstrated a sizable proportion of missing prescriptions in 2017 and 2018 [2] This is likely to impact detection of long-term conditions that are managed within primary care, therefore this research operationalised a relaxed definition of 2 prescriptions instead of 4 in any 12-month period which had been used in other studies [Lee et al].[3]

When using either secondary care diagnoses or community medications to study long-term conditions researchers should consider changes over time in service delivery which could potentially impact on detection rates within routinely collected data. For example, the decline of detection rates for dermatological conditions may reflect a reduction in inpatient services in the region [4] It is also possible that improvements in clinical coding for hospital admissions and improved ingestion rates of prescription items into EPD may have contributed to the upward trends in prevalence of multimorbidity observed in 2012-13 in the present study. In addition, the observable drop in the prevalence of maternal multimorbidity in 2020 across most groups may be explained by the emergence of the COVID-19 pandemic. It is possible that during this time, women with pre-existing physical and mental health conditions had different reproductive choices and behaviours, as well as reduced access to fertility services.[5] Future epidemiological study of long-term conditions should also take account of the impact of the pandemic and related restrictions on hospital admissions, and hence secondary care ICD-10 diagnostic codes pertaining to long-term health conditions. For the present study, however, it is unlikely to have had a significant impact as the cohort included women whose pregnancies commenced before or during 2020 and therefore rely on look-back periods prior to restrictions for detection of long-term conditions.

**References:**

1. MacRae C, Mercer SW, Henderson D, McMinn M, Morales DR, Jefferson E, et al. Age, sex, and socioeconomic differences in multimorbidity measured in four ways: UK primary care cross-sectional analysis. British Journal of General Practice 2023; 73 (729): e249-e256; doi: 10.3399/BJGP.2022.0405.
2. Maguire A, Kent L, O'Neill S, O'Hagan D, O'Reilly D. Impact of the COVID-19 pandemic on psychotropic medication uptake: time-series analysis of a population-wide cohort. Br J Psychiatry. 2022 Dec;221(6):748-757; doi:10.1192/bjp.2022.112
3. Lee SI, Azcoaga-Lorenzo A, Agrawal U, Kennedy JI, Fagbamigbe AF, Hope H, et al. Epidemiology of pre-existing multimorbidity in pregnant women in the UK in 2018: a population-based cross-sectional study. BMC Pregnancy and Childbirth 2022;22:120; doi: https://doi.org/10.1186/s12884-022-04442-3.
4. British Association of Dermatologists. Dermatology Inpatient Consultation Report 2022. <https://cdn.bad.org.uk/uploads/2022/11/16110400/Dermatology-Inpatient-Consultation-Report_Nov_22.pdf>
5. Flynn AC, Kavanagh K, Smith AD, Poston L, White SL. The Impact of the COVID-19 Pandemic on Pregnancy Planning Behaviors. Womens Health Rep (New Rochelle). 2021 Mar 23;2(1):71-77; doi: 10.1089/whr.2021.0005.
